# Advancement Opportunities and Endeavor of Innovative Targeted Therapies for Small Cell Lung Cancer

**DOI:** 10.7150/ijbs.105973

**Published:** 2025-01-20

**Authors:** Wei Ouyang, Ziyao Xu, Shaoyu Guan, Yang Hu, Xiaoxue Gou, Zhe Liu, Wei Guo, Ye Huang, Lifen Zhang, Xingmei Zhang, Tian Li, Bin Yang

**Affiliations:** 1 Hubei Cancer Hospital, Tongji Medical College, Huazhong University of science and Technology, Wuhan, Hubei, China.; 2 Department of General Surgery, The first Medical Center of Chinese PLA General Hospital, No. 28 Fuxing Road, Beijing 100853, China.; 3 Pharmaceutical Sciences Research Division, Department of Pharmacy, Medical Supplies Centre of PLA General Hospital/Medical School of Chinese PLA, Beijing 100853, China.; 4 Department of Oncology, Yichang Central People's Hospital, Yichang, Hubei, China.; 5 Department of Pancreatic-Biliary Surgery, The First Hospital of China Medical University, Shenyang, Liaoning 110001, China.; 6 Department of Respiratory Medicine, Enshi Central Hospital, Enshi 445000, Hubei, China.; 7 Department of Oncology, The First Affiliated Hospital of Xi'an Jiaotong University, Xi'an 710061, China.; 8 College of Medical Technology, Chengdu University of Traditional Chinese Medicine, Chengdu 610000, China.; 9 Tianjin Medical University, Tianjin 300100, China.

**Keywords:** Small Cell Lung Cancer, Targeted Therapy, Delta-like ligand 3, Chimeric antigen receptor T, Antibody-drug conjugates

## Abstract

Small cell lung cancer (SCLC) is an intractable disease with rapid progression and high mortality, presenting a persistent obstacle impeding clinical management. Although recent advancements in immunotherapy have enhanced the response rates of platinum-based chemotherapy regimens, the emergence of acquired resistance invariably leads to recurrence and metastasis. Consequently, there is an urgent necessity to explore novel therapeutic targets and optimize existing treatment strategies. This article comprehensively reviews the currently available therapeutic modalities for SCLC. It delves into the immunologic prognostic implications by analyzing selected immune-related signatures. Moreover, it conducts an in-depth exploration of the molecular subtyping of SCLC and the associated molecular pathways to identify potential therapeutic targets. Specifically, the focus is on clinical interventions targeting delta-like ligand 3 (DLL3), elucidating its resistance mechanisms and demonstrating its notable antitumor efficacy. Furthermore, the study examines the mechanisms of chimeric antigen receptor (CAR) T and antibody-drug conjugate (ADC), covering resistance issues and strategies for optimizing resistance management, with particular emphasis being placed on analyzing the prospects and clinical value of CAR T therapy in the context of SCLC. Moreover, the effectiveness of poly ADP-ribose polymerase and ataxia telangiectasia and rad3/checkpoint kinase 1 inhibitors is discussed and underscores the advantages of combining these inhibitors with standard chemotherapy to combat chemoresistance and enhance the antitumor effects of immunotherapies. Overall, this study investigates emerging strategies for targeted therapies and optimized combination regimens to overcome resistance in SCLC and highlights future strategies for new therapeutic technologies for SCLC.

## 1. Introduction

Lung cancer remains a major killer worldwide^1^-^5^. Small cell lung cancer (SCLC) represents a highly aggressive and treatment-resistant form of lung cancer that occurs in roughly 15% of all cases^6^, ^7^. It is estimated that 250,000 new cases of SCLC worldwide occur annually, resulting in up to 200,000 mortalities^8^. SCLC onset predominates in individuals over the age of 50, with a slightly higher incidence in males compared to females, yet with a decreasing gap among the genders. Furthermore, the morbidity associated with SCLC involves hazardous factors like tobacco and environmental pollution, where duration and magnitude of smoking are favorably correlated with morbidity prevalence^9^, ^10^. Geographic and racial disparities contribute to variations in the prevalence of SCLC, with African Americans exhibiting a higher incidence compared to Caucasians, and similarly, the rate of SCLC is descending in areas of high-income regions and countries^11^-^14^. SCLC is characterized by rapid prolongation with early dissemination of metastases, leading to nearly 70% of cases with remote metastases at the time of diagnosis, resulting in a 5-year survival rate of a mere 6.4%, in contrast to the 25-33% survival rate among patients diagnosed at an early stage^15^. Emphasizing the pressing necessity for the advancement of pharmaceuticals and therapeutic approaches for SCLC based on the current epidemiological status quo.

American Joint Committee on Cancer tumor-nodes-metastasis (TNM) staging manual commonly utilized for non-small cell lung cancer can also be effectively applied to the staging of SCLC. Yet staging systems for SCLC that are adopted more frequently adopt to a simplified binary staging system proposed by the Lung Cancer Study Group of the Veterans' Administration, dividing SCLC into a limited-stage (LS) and an extensive-stage (ES). LS-SCLC is confined to a single side of the thorax, whereas ES-SCLC is anywhere beyond the limits of the limited stage and corresponds to stage IV disease in the TNM staging system. Given the limited advances available in the current treatment paradigm, and the fact that overall survival (OS) of patients is alarming whether LS-SCLC or ES-SCLC, innovative therapeutic alternatives with improved efficacy warrant exploration regarding the treatment of SCLC in general, and ES-SCLC in particular.

## 2. Standard Treatment Models and Biomarker Exploration

### 2.1 Strategies under different stages of SCLC management

Standard management strategies across various stages of SCLC currently pose remarkable variations as well^16^: In cases of LS-SCLC, the primary treatment modalities typically include sequential or simultaneous radiochemotherapy, with radical surgery being considered for a minority of early cases. Etoposide-cisplatin remains the most commonly used initial treatment regimen. In addition, radiotherapy is crucial during the early stages to augment the survival and local control rates in patients with LS-SCLC. Prophylactic cranial irradiation in LS-SCLC patients who have achieved remission following concurrent chemoradiotherapy shows promise in reducing the incidence of intracranial metastases and enhancing OS rates. On top of that, the new paradigm of adding Duvarizumab adjuvant therapy followed by simultaneous radiotherapy reformulates the status quo of LS-SCLC management and prolongs OS and progression free survival notably in patients with LS-SCLC^17^. In contrast, systemic therapy remains the established treatment modality for ES-SCLC, with localized radiation therapy offering potential benefits in terms of antitumor efficacy. Amidst the current status quo of a first-line treatment strategy that traditionally utilizes platinum-based dual-agent chemotherapy with etoposide or irinotecan with marginal benefit to patients, with the major breakthroughs achieved by combining produced programmed death-1 (PD-1) / programmed death ligand 1 (PD-L1) immune checkpoint inhibitors (ICIs) (e.g., Atezolizumab and Durvalumab), and subsequent results of domestically produced PD-1 / PD-L1 inhibitor (e.g., Adebrelimab, Serplulimab, Toripalimab and Tirelizumab) demonstrating similar efficacy to existing immunotherapies, combat therapies markedly improved survival prognosis for patients with ES-SCLC via synergistic effects as well as the advantage of elevated chemo sensitivity, thus augmenting the standard dual-agent chemotherapy regimen for SCLC^18^, ^19^. Further innovation was recorded in the combination of Bemosuzumab (a humanised IgG1 subtype PD-L1 inhibitor) with chemotherapy-added Anilotinib (a multi-targeted anti-angiogenic small molecule) to achieve a new milestone in OS, with a duration of 19.3 versus 11.9 months in the control group (hazard ratio 0.61; *P* = 0.0002)^20^, derivation of an immuno-combination based on the anti-angiogenic effect on the anti-tumor synergy theories^21^, ^22^.

Among the backline treatments, Topotecan is the second-line drug approved by the U.S. Food and Drug Administration (FDA) for the treatment of SCLC. And Lurbinectedin, a selective oncogenic transcriptional inhibitor, got approved as a second-line drug for metastatic SCLC^23^. More recently Tarlatamab that engages bispecific T-cell engagers (BiTEs) targeting the delta-like ligand 3 (DLL3) stellar molecule remediated the SCLC backline treatment dilemma with accelerated FDA approval by revealing prominent anti-tumor activity and objective remission rates (ORR), notably observed in patients with brain metastases with potential beneficial value^24^, which would be focused on later in the overview. Within the immunotherapy field, Pembrolizumab yielded an ORR of 19.3% and a median OS of 7.7 months in patients with advanced SCLC in the KEYNOTE-028 and KEYNOTE-158 studies^25^, while Nivolumab alone and in combination with Nivolumab with Ipilimumab have also shown efficacy in treating SCLC, with ORR of 11.6% and 21.9% respectively in the CheckMate032 study^26^. Apart by ICIs that target multi-target anti-angiogenesis agents, Anilotinib has been approved as proposed for third-line and upward therapies for ES-SCLC of China upon favorable clinical data and safety profile^27^. Although demonstrating clinical efficacy in drug-resistant diseases, this has not resulted in a substantial improvement in OS. Regarding further backline therapies, approved ICIs (Pembrolizumab and Nivolumab), targeted therapies, and anti-vascular combination ICIs have enhanced drug efficacy by modulating the tumor microenvironment. In addition, recent results in ongoing research areas such as ICIs, antigen-specific vaccines and tumor vaccines are showing promise and are emerging as focal points in the research of SCLC treatment (**Table 1**).

### 2.2 Assessment of biomarkers for efficacy prediction

The combination of ICIs has demonstrated enhanced patient survival and improved outcomes^28^-^31^. however, the efficacy of ICIs therapies remains constrained, hindering widespread adoption of breakthroughs. The obstacles include insufficient benefit, paucity of recipients, and unavailability of predictive biomarkers impede progress. Particularly, the deficiency in identifying key biomarkers and novel therapeutic targets associated with treatment response poses a major dilemma limiting the development of existing therapeutic strategies. Firstly, regarding the exploration of ICIs-associated biomarkers to predict the efficacy and prognosis of SCLC, although ICIs serves as the prevailing standard of care for SCLC, real-world studies indicated that the sustained OS benefit of ICIs are confined to a minority of patients^30^, and thus the exploration of novel clinical ICIs-associated biomarkers for predicting the beneficiary patient would be of notable clinical merit. Nonetheless ICI-related predictive biomarkers remain elusive.

Due to its strong association with tobacco exposure, SCLC typically exhibits extensive genomic alterations, such as mutations, insertions, deletions, copy number alterations, and chromosomal rearrangements, etc., leading to a high tumor mutational (TMB), and causing possession of a greater diversity of neoantigens^32^, ^33^. The elevated neoantigen production resulting from TMB enhances presentation to T cells, thereby strengthening the immune response. Thus, acting as a surrogate for neoantigen generation in tumor cells, TMB is often utilized to predict the potential of the host immune system to recognize the peptide as an exogenous antigen and to initiate a cytotoxic response. TMB is defined by the number of non-synonymous mutations per megabase (mut/Mb) in somatic cells within a given region and is usually expressed in terms of the number of mutations per Mb^34^. The evaluation of TMB is influenced by various factors, including the sample quality and volume, the size of the tested genome, and the method of bioinformatics analysis. The thresholds and predictive value of TMB obtained with different testing assays ought to be systematically evaluated to judge compatibility. The absence of a standardized and precise definition of high TMB thresholds currently complicates the establishment of a specific critical value for predictive purposes. In the field of lung cancer, the median TMB of non-squamous non-small cell lung cancer is approximately 8mut/Mb, while that of SCLC is around 10mut/Mb^35^. However, contrary to non-small cell lung cancer where high anti-tumor activity exists for patients with high TMB, ICIs have failed notable benefits for SCLC. In 2020, FDA approved pembrolizumab for the treatment of adults with high tumor tissue mutational load (tTMB-H: defined as ≥10mut/Mb in unresectable or metastatic solid tumors) in adult and pediatric patients), which enrolled patients with 10 advanced unresectable solid tumors including SCLC. 34 SCLC patients were tTMB-H. The ORR for tTMB-H and non-tTMB-H SCLC patients were 29% and 9.5%, respectively, with a median OS of 9.4 (95% CI: 5.6-19.1) and 6.3 (95% CI: 3.9-7.7) months. Similarly, in the Checkmate 032 study, patients with tumors with high TMB were more sensitive to the combination of Nivolumab or Ipilimumab combined with Nivolumab^36^. Further analysis in the IMpower133 study revealed that the results of blood-based TMB-H (10 or 16 mut/Mb) were not predictive for the efficacy of Atezolizumab combination chemotherapy in ES-SCLC^18^. Thus, the utility of TMB as a predictive biomarker of response to ICIs in SCLC remains debatable.

PD-L1 protein expression has become a crucial biomarker for assessing the effectiveness of ICIs in clinical settings and is now utilized to inform treatment decisions regarding ICIs. Each of the currently approved ICI drugs is accompanied by a corresponding diagnostic method utilizing PD-L1 immunohistochemistry. Yet, thresholds for immunohistochemical testing and defining positive results have not yet been subject to harmonization across reagent testing products. PD-L1 expression was predominantly low in SCLC, where 98% of patients showed PD-L1 expression levels <5% in SCLC tumor cells^37^. Additionally, ICIs have provided only limited long-term benefits for SCLC patients compared to non-small cell lung cancer. Neither the results of the follow-up analysis of the CheckMate 032 study nor the exploratory analysis of the IMpower133 study demonstrated a PD-L1-dependent clinical benefit in patients treated with Nivolumab or Atezolizumab. Therefore, based on the current data, it is suggested that PD-L1 expression status is inaccurate in predicting patient outcomes despite the fact that a minority of patients have improved ORR in combination with ICIs^18^, ^38^. As previously described, with the revelation that different SCLC molecular subtypes also correlate with ICIs sensitivity profiles, SCLC with low neuroendocrine (NE) expression, with higher proportions of cytotoxic T-cells, natural killer cells, and macrophages as well as higher expression of immune checkpoint molecules versus high NE SCLC patients, exhibited a superior efficacy for ICIs. These findings corroborated by the IMpower133 trial, which showed a significant survival benefit in SCLC type I patients. Since high PD-L1 expression generally will not overlap appreciably with TMB-H, it is possible that a combination based on PD-L1 expression and TMB may offer somewhat better prediction than the sole utilizing of a solitary biomarker in practice^39^. The effects of such parameters as the presence of temporal or spatial heterogeneity of PD-L1 expression, tumor area and allocation of PD-L1 expression need for comprehensive consideration.

Profiling of treatment vulnerability based on SCLC molecular subtypes (see SCLC typing described below), coupled with clinical data from Impower133 revealing a defined OS benefit in the SCIC-I subtype, indicating that the SCLC-I subtype represents a promising migrant biomarker for predicting immune response. Other potential immune-related biomarkers under investigation include the presence of specific immune cell subsets within the tumor microenvironment and the expression of other ICIs such as Cytotoxic T lymphocyte-associated protein 4. Apart from the commonly used clinical biomarkers, regarding the potential reasons for the poor efficacy of ICIs, relevant studies indicated that which may also include factors such as Several factors also contribute to the immune response process: low tumor-infiltrating lymphocyte (TIL) counts, low expression of major histocompatibility complex (MHC) class, presence of immunosuppressive cell populations and cytokines, and the presence of avascular tumor areas from rapid tumor growth to evade immunity. Previous studies revealed that MHC-I is generally lowly expressed in SCLC with increased expression of MHC-I recruiting and a significant increase in CD8^+^ T cells resulting in increased anti-tumor response to ICIs. Researchers categorized MHC-I expression levels in SCLC patients who benefited from ICIs and found a positive association between the level of expression and the duration of response to ICIs. This indicates that the MHC-I expression profile could act as a potential biomarker for SCLC immunotherapy^40^, ^41^. It was observed that the MHC-II gene possessed different immunogenicity from MHC-I in the mouse model, deficiency of the MHC-II gene also affected the anti-tumor response. Besides, MHC-II inhibited CTLA-4 and promoted the efficacy of ICIs^42^. These results implied that MHC I/II might help to evaluate the immune response to ICIs. Analysis of multitude of studies indicating that patients with higher TIL counts or the presence of paraneoplastic neurological syndrome (PNS) were associated with a favorable prognosis^43^, revealing a possible correlation with tumor tissues from patients with SCLC displaying an increased interplay between TIL and PD-1/PD-L1, as well as tumor cellular antibody mechanisms, thus supporting the potential of these metrics to be used as prognostic biomarkers. The subtype of lung cancer known to be most frequently associated with PNS occurred in SCLC, with approximately 10% of patients presenting PNS to some extent. Moreover, activation of Notch signaling correlate with an increase in intrinsic tumor immunity, a finding that provides support for the potential use of Notch as signatures for predicting a durable response to immunotherapy in SCLC. And finally peripheral circulating tumor DNA mutations were tested by liquid biopsy as predictors of efficacy post immunotherapy in SCLC. Circulating tumor DNA molecules will remain an appreciable correlation predictor of OS^44^, yet sufficient samples are still needed for evaluation. A robust biomarker for prediction on the SCLC immunotherapy response pending as of today, irrespective of the initial efforts to identify aforementioned markers. Future work aimed at deepening the understanding of the immune profile of SCLC is valued for clinical guidance in optimizing immunotherapy strategies and probing for effective biomarkers.

Among those potential therapeutic targets for SCLC, Schlafen family member 11 (SLFN11), a DNA/RNA deconjugating enzyme that induces irreversible replication blockade, several clinical studies have revealed that SLFN11 has the potential to predict the role of inhibitors of DNA damage repair, such as poly ADP-ribose polymerase (PARP) inhibitors biomarkers in a variety of tumor types including SCLC, and in subsequently conducted clinical studies about the combination therapy group containing PARP inhibitors for the treatment of recurrent SCLC, it was observed that SLFN11-positive patients performed more favorable PFS and OS to negative patients notably, further speculating that SLFN11 has the potential to prevent the efficacy of PARP inhibitors in SCLC value. In a similar vein, DLL3 mentioned above functions as a NOTCH-primed heterogeneous ligand, which enables its endless potential for SCLC treatment and efficacy prediction attributed to its specific expression profile on the surface of SCLC cells (which will be elaborated in detail below).

Although the above biomarkers may possess promising potency for the prediction of SCLC efficacy, stratified analyses relying on molecular subtypes and genomic patterns may render reliable value for information such as efficacy assessment and survival in SCLC. Also to be considered is the point that currently available these biomarkers have only ever been evaluated in minor cohorts and no reliable conclusions that confirm either prognostic or predictive value need to be drawn. Furthermore, the highly heterogeneous nature of SCLC may limit the accuracy of a solitary biomarker, thus coming studies possibly devoted to the integration of multiple factors for the generation of candidate biomarkers and to finding novice complementary pathways to augment the antitumor response.

## 3. Pathologic and molecular subtyping of SCLC

The pervasive deactivation of crucial suppressor oncogenes TP53 and RB1, coupled with NE/epithelial differentiation, has permitted in the widespread consensus in the molecular homogeneity of SCLC^45^. Beginning in the 1980s, investigators categorized SCLC into classic and variant phenotypes according to the differential expression of genes related to NE proteins^46^. The classic phenotype, representing approximately 70% of cell lines, is characterized by densely clustered growth and high expression of NE-related proteins. The variant phenotype further divides into morphological and chemical subtypes, with the former exhibiting cell adhesion to culture dishes and the latter showing tight cell clusters with a decreased expression of NE-related proteins^47^. Subsequent research re-categorized the classic subtype into ASCL1, characterized by high levels of ASCL1 and NE markers, while the variant subtype with elevated NEUROD1 exhibits high NEUROD1 expression and a partial or complete absence of certain NE proteins. Currently, the definition of SCLC subtypes has evolved from classic/variant to NE/non-NE, further transitioning to molecular subtypes delineated by transcription factors. The speedy development of molecular and cellular biology would extricate SCLC from this terrain of difficulty in perceiving the criteria for histologic stratification of SCLC. Previously investors had initially identified four main molecular subtypes of SCLC by the expression levels of transcription factors in recent years: ASCL1 (SCLC-A), NEUROD1 (SCLC-N), POU2F3 (SCLC-P), and YAP1 (SCLC-Y)^48^, ^49^. The majority of SCLC was found to express ASCL1 as depicted by the analysis of gene expression levels, with one report depicting that ASCL1 exhibited in about 70% of SCLC cases, compared to ~11% for NEUROD1. Expression of POU2F3 in about 16% of SCLC cases was observed via gene expression, and in about 7% at the protein level, with levels repugnant to ASCL1 and NEUROD1^47^. Notably, these isoforms are not co-segregated with specific molecular alterations (e.g., P53, Rb, etc.) and harbor distinctive distinguishing features and common characteristics. SCLC-A and SCLC-N are characterized by increased NE phenotypes, high levels of NE markers (synaptophysin, chromogranin A, etc.), and high DLL3 expression compared to SCLC-P subtypes^50^. Nevertheless, the following studies raised queries about the expression or relevance of YAP1 for the YAP1 isoforms. Analysis of the initial gene expression revealed that YAP1 was expressed in only about 2% of SCLCs, while subsequent studies implied that YAP1 was expressed in about 10% of YAP1-positive SCLCs, and notably, analysis of the YAP1 mRNA showed that YAP1 mRNA expression failed to discriminate between distinct tumor subsets different from the ASCL1, NEUROD1, and POU2F3 subsets. Furthermore, large cohort studies demonstrate that YAP1 fails to identify SCLC-Y subtypes due to low expression levels in each subtype. Hence, Gay *et al.*^51^ redefined a novel subtype SCLC-I in 2021, which was based on the identification of SCLC-A, SCLC-N, and SCLC-P subtypes, with its low expression at the level of the three transcription factors at all but the expression of a variety of immune checkpoints, human leukocyte antigen (HLA) genes, as well as the characterization of having a high degree of immune cellular infiltration, referred to as the inflammatory subtype as well. Analysis in the Impower133 study demonstrated that of the four SCLC molecular subtypes, the SCLC-I subtype tended to offer superior benefits from immune checkpoint inhibitors^51^. However, SCLC-I exhibited the highest epithelial mesenchymal transition (EMT) characteristics, while SCLC-A had the relatively lowest EMT trend, suggesting that SCLC-I may be associated with early metastasis and the development of acquired resistance^52^. However, the SCLC-P tumor subtype is associated with the poorest benefit in contrast, irrespective of the combination of treatment modalities. Current available evidence supports specific therapeutic fragility for each SCLC subtype^49^, ranging from the potential efficacy of SCLC-A against B-cell lymphoma 2 (BCL-2) inhibitors and agents of DLL3, to the benefit of SCLC-N towards aurora kinase inhibitors and SCLC-P to PARP inhibitors, antimetabolite agents, and/or laser kinase inhibitors^53^-^55^. Furthermore, LKB1/STK11 mutation might potentially serve as a subtyping indicator for SCLC as well^56^. The following section underscores the emerging therapeutic targets, potential molecular mechanisms, and efficacy observed in preclinical and clinical studies.

## 4. Investigating mechanisms of drug resistance in SCLC

Despite the fact that prevailing therapeutic strategies in SCLC have yielded improved survival prognosis for patients, and neoplastic therapies are rapidly progressing, the availability of SCLC treatments remains finite, rendering substantial advances in terms of efficacy and survival benefits elusive. Underlying factors involve the fact that almost all SCLC cases progress to disease recurrence owing to the development of refractory drugs following a period of treatment. In recurrent disease, chemotherapy proves to be ineffective and immunotherapy is not yet a viable option, so conquering tumor resistance presents an obstacle to improving the prognosis of patients. The mechanisms responsible for drug resistance in SCLC are mainly summarized as follows: The key molecular pathways involved in the transformation of SCLC to a NE-low phenotype include YAP1, NOTCH, Wnt family (WNT), and MYC signaling, all of which contribute to drug resistance in SCLC. Studies reveal that high expression of MYC family proteins induces chemotherapy resistance in SCLC in both *in vivo* and *in vitro* settings^28^, ^57^, ^58^. The YAP protein also mediates resistance to chemotherapy and radiotherapy via Notch-dependent and independent signaling pathways. Activation of the Notch pathway results in a transition from a NE to a non-NE state, thereby enhancing chemotherapy resistance in SCLC MYC family proteins can activate NOTCH signaling, further driving tumor subtype transformation and promoting resistance^59^-^61^. Findings also revealed that ASCL1 expression was decreased dramatically in chemotherapy-resistant and post-relapse cell lines, and the above studies implicate a mechanism of action between ASCL1 and non-NE or MYC expression in relation to chemotherapy-acquired resistance.

The growth factor signaling pathways, specifically the PI3K/AKT/mTOR pathways, play a crucial role in SCLC resistance mechanisms. Researchers observed, through gene and transcriptome data analysis, that PI3K/AKT and mTOR signaling are upregulated in chemotherapy-resistant SCLC cell lines and models. These pathways can be blocked by corresponding growth factor inhibitors, suggesting the potential of these inhibitors to reverse SCLC resistance. Clinical trials targeting relapsed SCLC patients are underway; however, the selection of treatment modalities requires further exploration. Additionally, studies confirm that DNA damage repair and apoptosis pathways contribute to SCLC resistance. Tumor cells enhance treatment resistance by upregulating apoptosis pathways, such as those involving BCL-2 protein. High expression of BCL-2 protein correlates with cisplatin resistance and poor prognosis. Inhibitors targeting BCL-2 protein can synergize with chemotherapy to promote apoptosis. Epigenetic factors, such as EZH2, transcriptional state alterations, and plasticity are potential mechanisms underlying SCLC resistance. EZH2 is upregulated in chemotherapy-resistant SCLC, promoting resistance through the epigenetic silencing of SLFN11. The efficacy of combining EZH2 inhibitors with chemotherapy in SCLC treatment is anticipated. Plasticity, the mechanism by which tumor cells acquire unique cellular characteristics, endows them with responsiveness to treatment and correlates with SCLC resistance. Studies show that tumor cell surface glycolipids and glycoproteins, involved in immune modulation, as well as T cell checkpoint molecules, such as B7-H3, play roles in immune suppression signaling.

## 5. DLL3-Targeted Therapies Research and Progress

**Figure 1:**
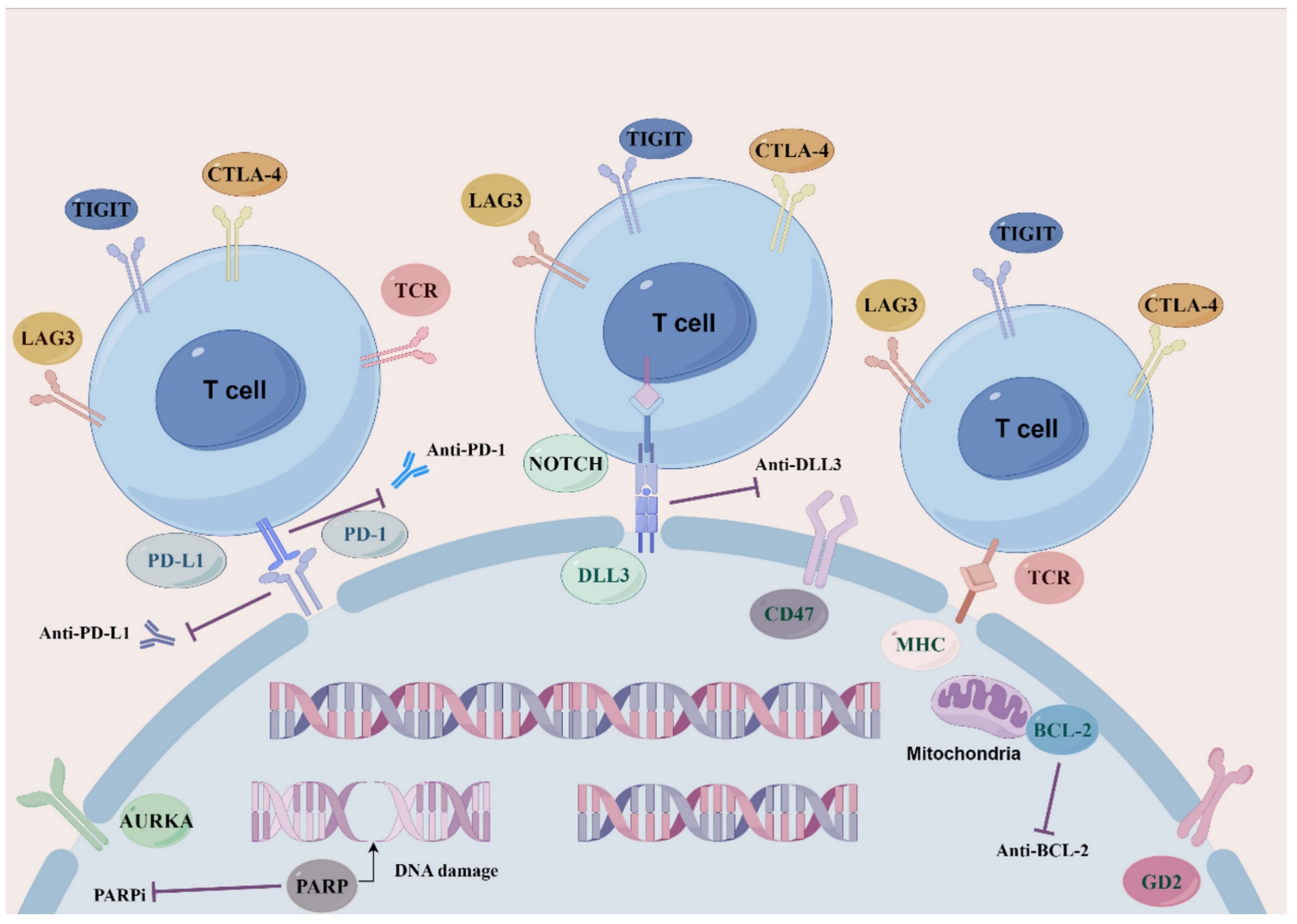
Overview of novel representative therapeutic targets for SCLC. Schematic diagram describes potential therapeutic targets to treat SCLC. including SCLC DLL3-specific antigens involved in ligands of NOTCH signaling pathway. BCL-2 family proteins that mediate apoptosis. Cell cycle and DNA damage repair pathways, PARP poly (ADP) -ribose polymerase, AURKA Aurora A kinase. Immune checkpoint molecules, PD-1, PD-L1, CTLA-4, TIGIT and LAG3.

As current single-agent and combination therapies for SCLC recurrence and drug resistance yield modest remission rates and survival benefits, new molecular pathways and targets along with other therapeutic strategies need compelling exploration and development so as to maximize the efficacy of SCLC regimens and to tackle the obstacle of drug resistance (**Figure 1: Novel target therapeutic mechanisms for SCLC**).

### 5.1 DLL3

As mentioned concisely above, substitutive strategies for SCLC are yet under exploration. Among participating in diverse SCLC-associated signaling pathways and targets, the Notch pathway is of great significance for the development of lung NE cells, together with proven tumor suppressor effects in NE tumors. Simultaneously, the Notch pathway has been shown to be involved in a variety of oncogenic processes in SCLC, including cell proliferation, differentiation, NE cell plasticity, acquired resistance to chemotherapeutic agents, and regulation of the tumor immune microenvironment. Among that DLL3 as a member of the Delta/serrated/Lag-2 ligand family, is an inhibitory ligand for tumor-specific surface antigens and the Notch signaling pathway in SCLC cells, encoding for inhibition of the activation of the Notch signaling pathway, thereby affecting tumorigenesis, progression, and/or chemoresistance. DLL3 with elevated cell surface specificity in SCLC and other NE tumors is rarely expressed or even omitted on normal cells, and its overexpression promotes the growth as well as the invasive and metastatic ability of SCLC cells. Notably, DLL3 is associated with the acquisition of resistance to platinum-based chemotherapeutic agents by promoting cell proliferation, with its elevated expression level also relevant to advanced staging and dismal prognosis of tumors^62^. Moreover, ASCL1 is a carcinogenic driver of SCLC^63^, driving SCLC disease progression and cell death by regulating the expression of multiple proto-oncogenes as well as DLL3^64^. As such, taking consideration of the mechanism of variable expression and the pathway of DLL3 in SCLC, targeting DLL3 as a selective therapeutic for SCLC emerged as a new direction in the current research field, and the development and assessment of DLL3-specific targeted SCLC therapeutics are being sought following by the researchers (**Table 2**).

### 5.2 DLL3-CAR T

#### 5.2.1 Principles and mechanisms of CAR T

Chimeric antigen receptor (CAR) T-cells are synthetic proteins engineered to target T cells against specific antigens on tumor cells to engender an anti-tumor immune response. CAR T has seen widespread implementation across hematological oncology fields, that the winningest CAR T target is the targeting of the B-cell antigen CD19, where reports showed sustained remissions achieved by CAR T proved commercially viable, which has become the standard of care for large B-cell lymphomas^65^, in addition to propelling therapies towards the solid tumors field in that long term. CAR T cells composed of three main components: the extracellular antigen-binding domain, the transmembrane structural domain, and the intracellular signal transduction region. Extracellular antigen-binding domains designed to re-target T cells to recognize tumor cells with cognate antigens, and the single-chain variable region (scFv) of antibody sources against specific tumor-associated antigens are modified and structured to become extracellular antigenic domains. Aiming at the expression of different tumor surface-specific antigens, researchers may formulate armed T cells that target antigenic molecules to achieving highly efficient tumor cell lethality. The hinge/transmembrane structural domain, connecting the extracellular antigen-binding domain to the intracellular signaling region, comprises homo- or heterodimeric membrane proteins, enabling modification of the degree of CAR gene expression by modifying the design of the transmembrane region. Among the intracellular signaling domains, the CD3ζ chain or immunoglobulin Fc receptor γ chain is the structural domain required for T-cell activation. The anti-tumor efficiency of T cells *in vivo* improved by the investigation and refinement of the structural domains of co-stimulatory molecules and co-stimulatory signaling molecules, which helped to stimulate the activation and value-added efficacy of T cells further and decelerated cell death. CAR serves as the pivotal segment within CAR T. It empowers T cells with the capability to acknowledge tumors in an HLA-independent mode, coupled with the advantage of utilizing natural T-cell expansion, lethality, and persistency. The assay is reliant on obtaining T cells from a patient or donors and utilizing genetic engineering techniques to modify a specific monoclonal antibody-derived scFv to act as an extracellular antigen-binding structural domain responsible for specifically recognizing and binding to tumor cell surface antigens. Then fused to a transmembrane chain segment consisting of a series of molecules involved in T-cell activation, such as CD28, 4-1BB and CD3ζ, the gene fragment was transmitted to the patient's peripheral blood T-cells through lentiviral or retroviral gene transduction, which became CAR T-cells expressing the CAR, and then returned to the patient's body after the *in vitro* CAR T-cells had been amplified to the desired therapeutic concentration (102 to 105-fold) for precision killing. The application of CAR T therapy has achieved notable success in inducing lasting remission in hematologic malignancies, which has sparked considerable enthusiasm for research in the realm of SCLC.

**Figure 2:**
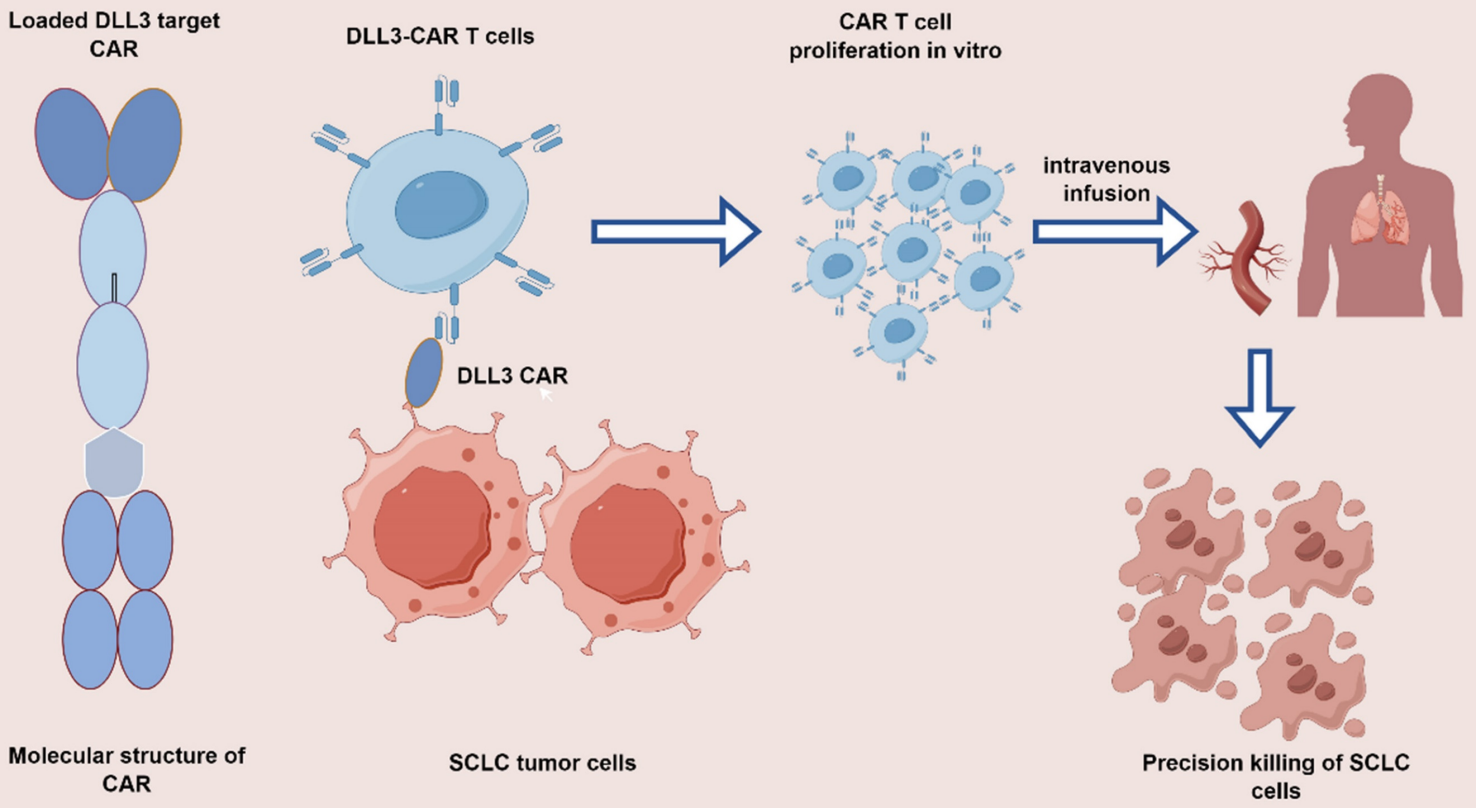
Mechanism of action of DLL3 CAR T-targeted drugs. Genetically modify patient-derived T cells *in vitro* to express CAR targeting DLL3, followed by extensive *in vitro* expansion. Redirect these cytotoxic T cells towards DLL3-SCLC cells to sustain and induce apoptosis in the SCLC cells.

As the forefront, amidst the equally hot field of CAR T therapy, the DLL3-CAR T therapeutic model holds much promise (**Figure 2**). Studies related to novel therapies for T cell retargeting targeting DLL3-CAR are ongoing in SCLC field^66^. AMG119 is a DLL3-CAR T-cell product consisting of a DLL3 antigen-binding domain-targeting, CD28, 4-1BB co-stimulatory binding domain-conjugated CD3 domain autologous inactivating lentiviral vector, which has shown long term anti-tumor activity in preclinical studies in DLL3-expressing SCLC cells. AMG19 also demonstrated comparable efficacy in relapsed/refractory SCLC patients in the I clinical trial (NCT03392064). Among combination strategies, a Phase I trial of AMG 119 (NCT03392064) involving tarlatamab is currently proceeding in anticipation of antitumor efficacy results^67^. An ongoing Phase 1, first-in-human study in ES-SCLC investigating LB2102 (DLL3 CAR T) (NCT05680922) and autologous T lymphocyte CAR cells against the GD2 antigen (iC9-GD2.CAR. IL-15 T-cells) among patients with advanced lung cancer, including SCLC (NCT05620342), CAR. T-cells showed a significant improvement in preclinical outcomes. (iC9-GD2.CAR IL-15 T-cells) in patients with advanced lung cancer including SCLC (NCT05620342), CAR T-cells have shown promising results in preclinical studies.

In addition, for a broad array of preclinical CAR T-cell products under development, all exhibited efficacy against DLL3-targeted tumor antigens. CAR T cells with the pro-inflammatory cytokine interleukin-18 (IL-18) significantly enhanced the efficacy of DLL3-targeted CAR T cell therapies in a SCLC model, and IL-18 also promoted the activation of CAR T cells and endogenous TILs in metastatic SCLC models in mice. IL-18 targeting DLL3-CAR T cells additionally exhibited an enhanced memory phenotype as well as induced durable responses, which synergized with the combination of anti-PD-1 agents. Besides, products including those with LB2102 and ALLO-213 are not yet available for clinical studies, with the expectation that the anti-tumor activity of the products will be further evaluated. The above findings indicated that DLL3 as an emerging potential target for SCLC cells, and the development of DLL3 CAR T-cells towards the anti-tumor activity of DLL3-positive SCLC potentially showed efficient persistence, specificity, and feasibility. CAR transduced natural killers (NK) cells (e.g., DLL3 CAR NK-92 cells), which complements CAR-T cell therapy have attracted attention toward tumor efficacy as configured with the NKG2D transmembrane structural domain and the co-stimulatory molecule 2B4-CD3 structural domain which might potentially enhance the cytotoxicity of NK cells, a preliminary anti-tumor activity in preclinical models with favorable tumor infiltration^68^, and the efficacy of CAR-NK in relapsed/refractory SCLC is being evaluated in a currently. A nascent cancer immunization technology, CAR T therapies exhibit multi-targeting properties with high affinity and specificity of the modified T cells through genetic engineering editing which is capable of recognizing a variety of identical antigens. Additionally, it is capable of massive expansion *in vivo* with the ability to preserve a sustained lethality and polarize into memory phenotype T cells, coupled with resistance to exhaustion. It features a universal protocol design that exceeds individualization limitations to achieve clinical scale-up. Nevertheless, the high mutation of tumors results in epitope heterogeneity, and intricate microenvironmental profiles of tumors impede the escape of tumor antigens. Particularly, the possibility of CAR T therapies triggering potentially adverse events (e.g., cytokine release syndrome, neurotoxicity, etc.), combined with production technology and cost issues including the availability of the technology in clinical practice, have all been identified to be impediments limiting its broader application.

#### 5.2.2 Mechanisms of CAR T-cell resistance

Retrofitted CAR assemblies confer on T cells the capability to specifically identify a broader range of target antigens than the natural T cell receptor. However, ongoing efforts also pose several challenges: limited efficacy in sustained remission, CAR T-cell resistance, and restricted drug accessibility. Thus, identifying the mechanisms associated with CAR T-cell dysfunction and overcoming CAR T-cell resistance is critical for generating CAR T-cells that remove tumor cells precisely and efficiently. The exploration of the mechanism of CAR T-cell resistance seems inevitable, and the following reasons may be considered for resisting CAR T-cells: 1) Resistance caused by antigenic modulation. Resistance caused by antigenic modulation. Antigenic regulation stands out as a critical cause of CAR T resistance in B-cell tumors and likewise poses a serious challenge in solid tumors. Owing to the prominent heterogeneity of most antigens, antigen density is modulated by a variety of mechanisms, encompassing genetic mutations, alternative RNA splicing, cellular lineage switching, epigenetic and/or post-transcriptional mechanisms that down-regulate antigen density and thereby decrease the CAR antigen density threshold, rendering the CAR incapable of full activation of the T-cells and leading to CAR T resistance. 2) Resistance caused by inadequate T-cell function. T-cell survival persistence, insufficient functional persistence and/or dysfunction have been linked to CAR T-cell resistance. Dysfunction commonly caused by T-cell depletion largely characterized by transcriptional and epigenetic reprogramming ultimately affecting terminal differentiation. Depletion of infused cells may also occur with CAR T transfusion back, or high tumor loads. 3) Drug resistance caused by impaired CAR T-cell transport and delivery. Transporting CAR T-cells to the tumor site to conjugate with surface target antigens provides a basic prerequisite for T-cell immunotherapy. Compared with hematological tumors, T-cell transport and delivery in solid tumors are usually limited by the immunosuppressive microenvironment, which prevents T-cells from being transported and infiltrated to the tumor site by secreting a number of chemokines, such as CXCL1, CXCL12, and CXCL5, and the T cells express the lack of corresponding chemokine receptors, thus severely limiting the potency of CAR T-cells to kill tumor cells. When CAR T-cells are transported to the tumor site, infiltration into the tumor microenvironment (TME) is a critical step to exert anti-tumor efficacy, while tumor-associated fibroblasts, myeloid cells forming extracellular matrix and other substances in the TME of solid tumors restrict the infiltration of T cells, organizing a continuous binding with tumor cells, which further reduces the anti-tumor efficacy of T cells. Impaired transport of CAR T-cells to the tumor site as described above is notably implicated in CAR T-cell resistance as well. 4) Resistance caused by the immunosuppressive state of TME. It is mentioned above that immunosuppressive TME exerts crucial effects on the differentiation and depletion of T-cell function, and the immunosuppressive microenvironment within solid tumors. Together with hypoxia, low pH, increased immunosuppressive cells (e.g., regulatory T-cells, myeloid-derived suppressor cells, and tumor-associated macrophages), inhibitory checkpoint ligands, and elevated production of tumor-derived cytokines (e.g., transforming growth factor-β), the combination of CAR and checkpoint blockade or depletion of other suppressive factors in the microenvironment contributes to the promotion of the T-cell exhaustion phenotype and to the resistance of CAR T-cells toward the tumor. 5) CAR T-cell immunogenicity. CAR T-cells harbor the propensity to induce anti-CAR cellular and humoral immune responses against non-self components of the CAR structure or residual proteins of gene transfer vector origin, which are intrinsically immunogenic and thus limit the efficacy of CAR T and inhibit sustained CAR T-cell responses.

#### 5.2.3 Strategies to optimize CAR T-cell therapy

Embarking on the improvement of T cell efficacy, specificity, safety and antigenic sensitivity as well as modulation of CAR signaling aimed at surmounting the resistance mechanisms of CAR T-cells could be a prospective new avenue. 1) Nano-based packaging and remote modulation of CAR T-cells. As a strategy designed to override immunosuppressive TME within solid tumors, the incorporation of nanotechnology enables control of immune cells with high spatiotemporal precision, accompanied by the ability to re-model TME. In particular, the alignment of nanotechnology with optogenetic instruments permits multifunctional spatiotemporal modulation of cellular activity. Thus, nanomaterials with stimulus conductivity allow remote control of cellular physiology through various technologies (e.g., near-infrared light, ultrasound, electromagnetism, and X-rays) with optogenetically based cellular regulation, facilitating precise and long-lasting tumor cell elimination via remote control of CAR T-cell therapeutics. Of the many modes of external stimulation, ultrasound stimulation offers the merits of fewer side effects and superior penetration. Ultrasound not only serves as a commonly available imaging and therapeutic tool in the medical and biological domains, as well as excelling in the activation and control of CAR gene expression on the surface of T cells. 2)The scope of CAR targets to target antigenic molecules expressed at low levels, thereby improving CAR molecular targeting sensitivity as well as specificity and expanding the range of CAR targets. Moreover, a potential drawback of CAR T cells compared to natural T cells concerns the inability to target intracellular antigens, as most of the abnormal proteins that drive cancer are found inside the cell. Researchers widened the shortcoming of targeting key oncogenic driver genes to improve CAR T targeting specificity by constructing prototype CAR molecules specific for peptides presented by MHC187 (pMHC), which allows the targeting of multiple HLA isoforms of pMHC using a PHOX2B peptide-MHC-specific scFv conjugate directed against the peptide that is overexpressed in neuroblastomas^69^. 3) Modification of CAR molecules and antigen screening. Similar to the continuous innovation of CAR molecular products, it is particularly crucial to screen and modulates CAR targets with a high degree of tumor specificity and coverage with respect to augmenting the anti-tumor efficacy and minimizing the toxicity of CAR T-cell therapies. One major obstacle in the current development of CAR T-cells for patients with solid tumors is that on-target but off-tumor toxicity (OTOT) resulting from the high risk of an inevitable complications due to the fact that the majority of candidate target antigens tend to be co-expressed on non-malignant tissues, aiming to conquer the off-target effect and enhance the antigenic specificity, sensitivity, and persistence. Preferably, the strategy of identifying desirable CAR molecules by means of a framework model via screening based on multi-omics data, high-throughput sequencing and integration in the screening of neoantigens for desirable targets is well-established. Multi-omics data from tumor and non-malignant tissues have provided a relatively potent library of candidate target antigens to support genome-wide screening of CAR targets in different tissues, cells, and specific cancer types. Candidate target antigens typically involve novel tumor-specific proteins, which are derived primarily from somatic mutations in tumor cells. To screen for neoantigens, comparative whole-exome or whole-genome sequencing of matched tumor and non-malignant samples is typically performed to identify tumor-specific non-synonymous somatic mutations, followed by RNA, protein sequencing, and mass spectrometry analyses to identify mutated expressed mRNAs and proteins. The development and widespread application of multi-omics technologies now provide a wealthy source of tumor-specific targets for screening novel ones.

Researchers have sought to tackle CAR T resistance and deficiencies by following threads. It is now recognized that the barriers of poor antigen targeting, the presence of extra-tumor toxicity, and resulting drug resistance that exist with a single CAR molecular target. So as to enable to overcome the deficiencies of a single target molecule, researchers have firstly reduced the problem of antigen escape by increasing the antigenic target, by the integration of two single-chain antibodies into a single receptor or dual CAR co-transduction of multiple CARs to administer a combination cell product that possess multi-specific CAR target antigens, and while still under evaluation in trials, the results of the pre-tests revealed security of multi-specific CAR molecules and held a prospect of improving anti-tumor efficacy by reducing antigen escape.

Researchers also tackled this problem with logic gated combinations of multiple CAR targets to improve tumor targeting specificity and reduce toxicity. Boolean logic gating with mathematical operators "IF/THEN", "AND", "OR" and "NOT " are applied to monitor the activation of CAR T-cells. In vulgar terms, CAR T-cells with logic gating are equivalent to installing different "logic switches" on tumor cells. For instance, CAR T-cells with "AND" gating refer to the activation of two target antigens that need to be expressed on tumor cells at the same time. CAR T-cells with "AND-NOT" logic gating can be activated only in case of the presence of the target antigen expressed on the tumor cells, while the antigen normally expressed on the non-malignant cells remains unavailable. This novel logic-gated approach holds the prospect of optimizing CAR T-cell design and enhancing the anti-tumor activity of CAR T-cells^70^, ^71^.

OTOT avoidance by modifying the scFv antibody in the CAR molecule in order to regulate the affinity for recognizing tumor cells together with decreasing the expression of tumor-associated antigens in non-tumor tissues. It has arisen as a potential new stratum to ameliorate the structure of the CAR molecule. CAR T-cells with high-affinity offer better response efficacy over tumor cells with low antigen expression density, yet could also resulting in the recognition of target antigens present in non-tumor tissues. Whereas low-affinity scFv antibody CAR T-cells lacked anti-tumor activity due to their inability to adequately recognize tumor cells with low levels of expression of tumor-associated antigens, it was also demonstrated that low-affinity scFv increased the risk of low-antigen density tumor cells evading detection and killing by CAR T-cells. Relevant studies in mice models have shown that OTOT is directly correlated with CAR affinity, with mice harboring CAR T-cells of high affinity exhibiting enhanced toxicity responses, while less damaging toxicity was observed with low affinity. In addition to modulating the scFv affinity of CAR molecules, development of novel receptors with low CAR antigen density thresholds represents an effective alternative. Modifications of the linkage and transmembrane regions (H/T) as well as adjusting the quantities of immunoreceptor tyrosine activation motif sequences (ITAMs) etc., for instance: decimation of ITAMs in the structural domains of CD3ζ would attenuate the cytotoxicity of CAR T-cells against cells of lower antigen densities but still maintain toxicity against cells of high antigenic target densities. The implication of this is of great reference for blocking immune evasion of low antigens and reducing cytotoxicity in parallel with inducing more potent anti-tumor activity^71^.

### 5.3 Anti-DLL3 ADC

#### 5.3.1 Principles and mechanisms of ADC

**Figure 3:**
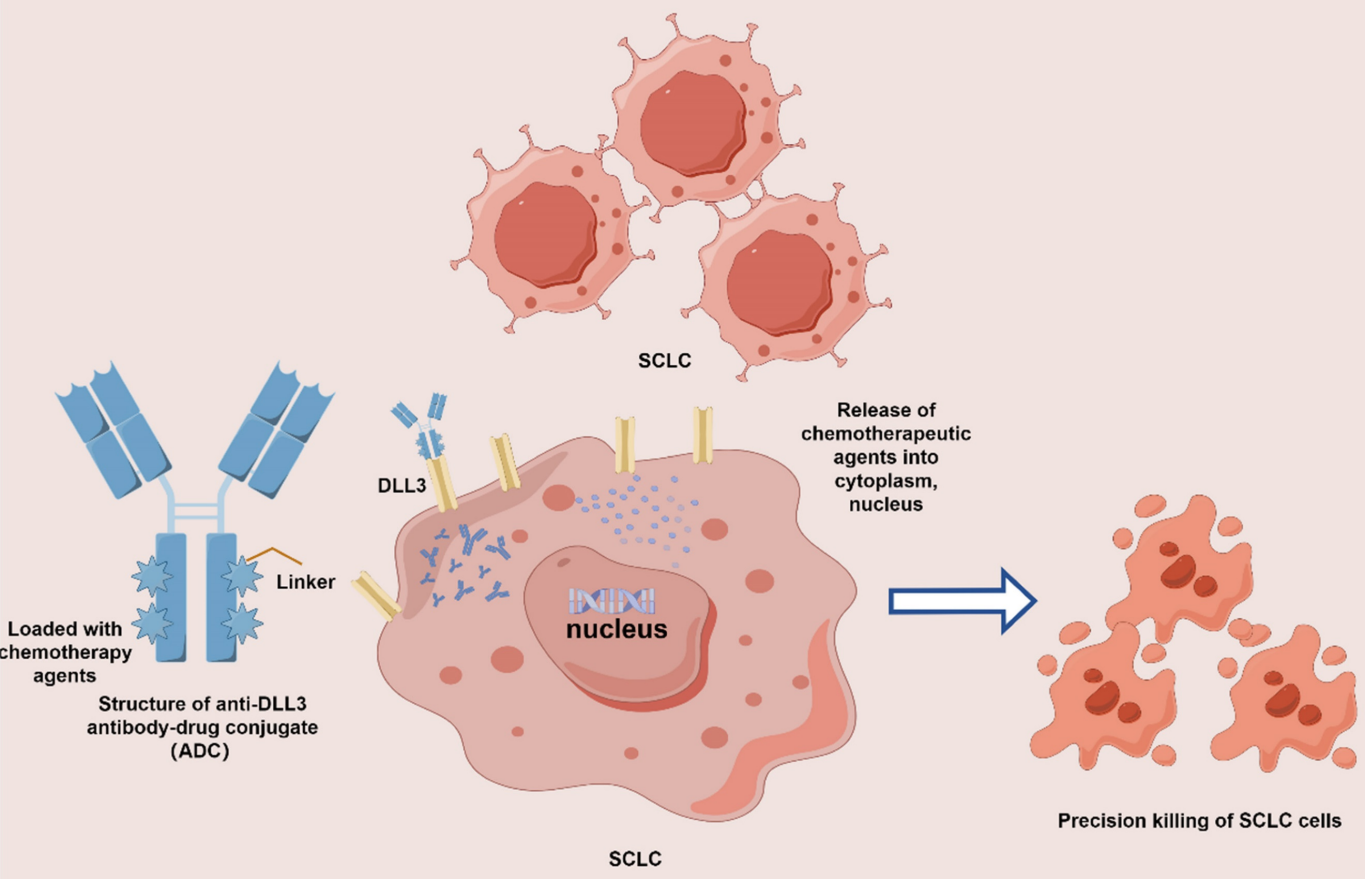
Mechanism of action of DLL3 antibody-drug conjugate (ADC). ADC comprises three primary components: an antibody targeted SCLC-associated antigen, a linker, and a cytotoxic payload. The mechanism of action for ADC in SCLC involves the monoclonal antibody binding to surface antigens on SCLC cells (e.g., DLL3). Following internalization into lysosomes, the linker is cleaved, releasing the cytotoxic payload, which results in targeted SCLC cells death.

Antibody-drug conjugates (ADCs) epitomize a substantial breakthrough in precision oncology, melding the precision of monoclonal antibodies with the lethal efficacy of chemotherapy drugs. Each ADC encompasses three principal elements: a targeted antibody, a potent cytotoxic agent, and a linker that unites them. The antibody is engineered to seek out antigens uniquely presented on cancer cells, facilitating the direct delivery of the toxic agent to the malignancy. This targeted approach aims to curtail systemic dissemination and mitigate adverse effects. The optimal antibody target is a cell-surface protein abundantly expressed by tumor cells yet sparingly on normal cells, thereby enabling more selective cytotoxicity while reducing systemic toxicities. The design of the linker must balance the need for stability to prevent off-target toxicity with the need for efficient release of the drug within the cancer. The cytotoxic payload in an ADC is the active component responsible for cell death upon internalization into cancer cells, primarily comprising potent microtubule inhibitors and DNA damaging agents. Ideally, the cytotoxic agents or payloads should be non-immunogenic, non-toxic during circulation in the bloodstream, and highly efficacious at sub-nanomolar concentrations. The objective is to broaden their therapeutic window by specifically targeting and delivering the drug to the cancerous cells.^72^ Upon intravenous administration, ADC selectively adhere to their designated antigens and are internalized via receptor-mediated endytosis. Subsequently, within the cellular milieu, the cytotoxic payload exerts its lethal effect through interference with either microtubules or DNA^73^ (**Figure 3**). Given the ADC-specific structure and potent anti-tumor activity, investigators are eagerly awaiting further studies and the results of ongoing trials to evaluate the efficacy and safety of ADCs to reshape the current therapeutic landscape of SCLC.

The development of ADCs has improved the therapeutic time window coupled with superior targeted properties. The anti-DLL3 ADC development has ventured forward with new orientations for SCLC. The first-in-class anti-DLL3 ADC, Rovalpituzumab tesirine (Rova-T), was designed to target and deliver cytotoxic agents to SCLC cells expressing DLL3. The antibody component of Rova-T binds to DLL3 on the surface of the cancer cells, while the conjugated cytotoxic drug is internalized and released within the cell, inducing apoptosis. Preclinical studies demonstrated that Rova-T exhibited potent and selective cytotoxicity against DLL3-expressing SCLC cell lines and xenograft models, providing the basis for subsequent clinical trials. Initial clinical trials investigating the efficacy of Rova-T in SCLC patients have shown promising results^74^. In phase I/II studies, Rova-T demonstrated a manageable safety profile and encouraged anti-tumor activity in patients with recurrent or refractory SCLC. In the phase III MERU study, maintenance therapy with increased Rova-T after first-line platinum-based chemotherapy yielded no improvement in OS compared with placebo (median OS 8.5 months with Rova-T *vs.* 9.8 months with placebo)^75^. And compared to topotecan as second-line therapy (TAHOE study) demonstrated no clinical advantage over standard treatment, while systemic drug-related toxicity events were observed in related studies. Considering that overall remission rates and remission durations varied and were poorly tolerated, this ultimately influenced the conduct of subsequent trials of Rova-T. The same targeted DLL3 ADC drug, known as SC002, hampered development in clinical studies against relapsed/refractory SCLC albeit with encouraging remissions, owing to its toxic side effects^76^. Another ADC containing a camptothecin derivative as payload targeting topoisomerase I and a methylsulfonyl pyrimidine tripeptide as cleavage linker, namely ZL-1310, awaits investigation to assess the safety, tolerability, and efficacy of ZL-1310 either alone or in combination with Atilizumab in patients with advanced SCLC following progression to platinum-based chemotherapy regimens (NCT06179069). However, the overall response rate and duration of response varied and with suboptimal tolerability, highlighting the need for further optimization of treatment protocols and patient selection criteria^77^-^79^.

#### 5.3.2 Mechanisms and optimization strategies for ADC resistance

Despite the initial promise of ADCs in the treatment of various malignancies, including SCLC, the development of resistance has become a substantial hurdle. This resistance can manifest at several junctures, encompassing ADC binding to the target antigen, compromised internalization of the ADC-antigen complex, modifications in intracellular trafficking, and shifts in the cancer cells' susceptibility to the cytotoxic payload. The primary manifestations of resistance to ADCs in cancer cells include: 1) Downregulation or loss of target antigens on the cell surface due to genetic mutations or selection of antigen-negative cell populations, compounded by tumor heterogeneity that may result in coexistence of antigen-positive and antigen-negative cells, thus impeding ADC efficacy. 2) Payload efficacy and toxicity: Tumor cells may develop resistance to cytotoxic drugs through various mechanisms. 3) Intracellular transport and processing: Alterations in the intracellular trafficking of the ADC-antigen complex can also lead to resistance. The potency of ADCs may be compromised if the conjugate fails to reach the appropriate intracellular location to release its cytotoxic payload, or if the linker is not effectively cleaved. 4) Antibody selection and optimization.^72^

To overcome the current resistance challenges and enhance ADC potency, it is essential to improve the current state through efforts including increasing the drug-to-antibody ratio, which should enhance ADC efficacy; developing new linkers and payloads less susceptible to resistance mechanisms or increasing the potency of the payload; selecting antibodies with high affinity and stability; and employing combination therapies that target multiple pathways.

### 5.4 DLL3-targeted BiTEs

BiTEs represent a novel category of synthetic bispecific monoclonal antibodies that exhibit considerable anti-neoplastic properties through the activation and targeting of T cells. BiTEs are characterized by a heightened specificity which enhances therapeutic efficacy and safety, simultaneously diminishing the adverse reactions commonly associated with monospecific antibodies. Comprising two ScFvs, BiTEs are designed to concurrently engage T cells and malignant cells. This interaction facilitates the formation of an immune synapse between T cells and the cancerous cells, thereby activating effector T cells in the tumor's vicinity and mobilizing a diverse array of immune cells to exert anti-tumor responses and eradicate cancer cells. Comparative studies with preclinical models have demonstrated that BiTEs possess superior anti-tumor capabilities when contrasted with traditional monoclonal antibodies and alternative bispecific antibody constructs. A BiTE that specifically targets DLL3 has the potential to selectively direct T cells against SCLC cells, thereby offering an innovative treatment modality that may overcome the constraints of conventional therapies and fulfill the substantial need for more effective SCLC treatments. Preliminary preclinical findings have indicated that DLL3-targeted BiTEs can effectively facilitate the engagement of T cells with SCLC cells, resulting in T cell activation, proliferation, and the ensuing destruction of SCLC cells both *in vitro* and *in vivo*. These promising results pave the way for the transition of DLL3-targeted BiTEs into early-phase clinical trials to ascertain their therapeutic potential in patients with SCLC.

Tarlatamab is a BiTE antibody that targets DLL3 on SCLC and the CD3 complex on T cells. Demonstrating significant therapeutic efficacy in early SCLC cell lines and patient-derived xenograft (PDX) mouse models^80^, Tarlatamab has exhibited preliminary antitumor activity in phase I clinical trials. The ORR was recorded at 23.4%, with 2 complete responses and 23 partial responses. The median PFS and OS were 3.7 months (95% CI: 2.1-5.4) and 13.2 months (95% CI: 10.5 to not reached), respectively. The most common treatment-related adverse event observed was cytokine release syndrome, affecting 52% of patients^81^. Toward the combination therapy arena, the recently announced results of the highlighted DeLLphi-303 study at the 2024 World Conference on Lung Cancer, a Phase IB study of first-line treatment for ES-SCLC aimed at evaluating the safety and efficacy of Tarlatamab in combination with a PD-L1 inhibitor administered with immunotherapy following combination immunotherapy as a standard first-line chemotherapy treatment, revealed that for Tarlatamab had a manageable safety profile and demonstrated sustained control rates when combined with the PD-L1 inhibitors Atezolizumab or Durvalumab as first-line maintenance therapy for ES-SCLC, with patients achieved a DCR of 62.5%, and a 9-month OS rate of 86.7% (combined with the Atezolizumab group) and 91.8% (combined with the Durvalumab group)^82^. In posterior therapies, the phase Ib (DeLLphi-302) study for ≥2 lines of treatment for SCLC was designed to evaluate the safety and tolerability of Tarlatamab combined with a PD-1 monoclonal antibody (AMG 404), with data expected to be published**.** Similarly, BI 764532 has shown effective antitumor activity in PDX mouse models^83^ and, in ongoing phase I studies of BI 764532 monotherapy for DLL3-positive SCLC, an ORR of 33% was noted among 24 patients receiving the target dose, along with manageable tolerability^84^. It was also in the area of combinations that the ongoing backline study of BI 764532 in combination with a PD-1 inhibitor (NCT05879978,) or with chemotherapy (NCT05990738) is aimed at evaluating the maximal tolerated dose and safety studies. Ongoing Phase I clinical trial (NCT05652686) designed to evaluate the efficacy of PT217in patients with advanced or refractory SCLC and other NE tumors expressing DLL3 by evaluating its Safety, Tolerability, Pharmacokinetic Parameters, and Preliminary Therapeutic Results targeted at DLL3 and CD47 Bites, indicated preliminary. Otherwise, agents, such as RO7616789, have demonstrated a positive anti-tumor signals, warranting additional assessment in clinical studies of their therapeutic efficacy. In other cases like domestic bispecific antibody targeting DLL3 and CD3 (QLS31904) has proved to exert marked inhibitory effects on tumor growth in preclinical models, with phase I studies (NCT05461287) evaluating the safety, tolerability, and pharmacokinetics of QLS31904 in patients with advanced solid tumors under active cultivation. Furthermore, the tri-specific T cell activating construct (TriTAC) HPN328, in interim data from phase I/II clinical trials (NCT04471727), has indicated favorable tolerability and clinical activity. ZG006 is targeted with a tri-specific antibody against CD3 and two distinct DLL3 epitopes. Prior to its clinical release this antibody caused significant tumor suppression in a mouse tumor model, which led to complete tumor regression in mice. ZG006 is currently undergoing multiple Phase 1 or Phase 1/2 clinical trial for the indication of SCLC or NE carcinoma (NCT06592638, NCT05978284, NCT06440057).

Some drawbacks of BiTE therapies deserve pondering which include short production half-life, possible induction of cytokine release syndrome and related adverse effects, deficiency of drug penetration, immunogenicity and inability to promote functional durability or prevent or rescue T-cells from exhaustion as well as resistance toward immune escape mechanisms of tumors. Related works are currently dedicated to ameliorating such limitations to enable a potentially broader and efficacious clinical application of BiTEs therapeutics.

## 6. Emerging Therapies

### 6.1 PARP inhibitors

SCLC normally exhibits a high expression of PARP, so therapeutic strategies developed against PARP inhibitors potentially offer new paradigms of drug-resistant treatment for SCLC. The safety of PARP inhibitors such as Olaparib, Rucaparib, or Talazoparib has been revealed in phase 1/2 clinical trials^85^.

### 6.2 Targeting the DNA Damage Response Pathway

The DNA Damage Response (DDR) pathway plays a pivotal role in detecting, signaling, and repairing DNA lesions, as well as regulating the cell cycle. However, aberrations in the DDR mechanisms are frequently associated with genomic instability and therapeutic resistance in various tumor cells, including SCLC. Moreover, studies have substantiated that the DDR is intricately involved in anti-tumor immune responses, targeting DNA damage responses and therefore positing DDR pathway targeting as a compelling strategy for sensitizing tumor cells to DNA-damaging agents, with potential implications for SCLC therapeutic approaches. Therapeutic strategies targeting DDR can be broadly categorized into two types: direct targeting of DDR proteins and exploitation of DDR deficiencies. Direct targeting involves the inhibition of key DDR proteins with pharmacological agents to impede the repair of DNA damage. Conversely, exploiting DDR deficiencies, such as synthetic lethality, entails the use of drugs that target compensatory repair pathways within cells deficient in specific DDR components, such as PARP inhibitors. PARP is an essential nuclear enzyme in the base excision repair (BER) pathway for single-strand DNA (ssDNA) break repair, and PARP inhibitors function by obstructing PARP-mediated DNA repair, particularly in SCLC tumors exhibiting defects in DNA repair pathways. Hence, PARP inhibitors have demonstrated therapeutic promise in the realm of targeted SCLC treatment. The efficacy of PARP inhibitors in SCLC has been evaluated in several clinical trials for combination therapy, albeit PARP inhibitors monotherapy has not observed favorable antitumor activity in SCLC. For instance, studies have indicated that a combination of Olaparib with temozolomide exhibits synergistic activity *in vivo* in recurrent SCLC, prolonging patient ORR and OS, and thereby enhancing antitumor activity^86^, ^87^. Additionally, studies have indicated that PARP inhibitors can elevate PD-L1 expression levels in SCLC models, thereby enhancing the antitumor immune response to ICIs. Consequently, the integration of PARP inhibitors with cytotoxic chemotherapy, other targeted agents (such as DDR inhibitors), and ICIs in combination therapies may enhance clinical outcomes and hold clinical significance for overcoming SCLC resistance to ICIs^88^.

Another salient feature of SCLC is the aberrantly elevated expression levels of DDR pathway mediators like the checkpoint kinase 1 (CHK1). Studies have indicated a significant upregulation of CHK1 mRNA levels in SCLC tumors compared to normal lung tissue, with CHK1 inhibition leading to increased DNA damage and cell death, especially in cancer cells already under replicative stress. Furthermore, the ataxia telangiectasia and rad3 (ATR)/CHK1 axis is part of a complex signaling network activated during genotoxic stress and DNA damage. Activation of ATR protein, upon interaction with ATRIP, leads to the phosphorylation and activation of multiple targets, including CHK1, ultimately arresting the cell cycle at the G2-M checkpoint until the damage is repaired. Consequently, targeting the heightened replicative stress response in SCLC cells via ATR inhibition holds potential for anti-tumor applications. Preclinical studies have confirmed that DDR pathway inhibitors exhibit significant antitumor activity, particularly in chemotherapy-resistant models. A plethora of ATR and CHK1 inhibitors have entered clinical trials for SCLC, being evaluated as monotherapies or in combination with chemotherapy. Prexasertib, a CHK1 inhibitor, was evaluated in a phase II study for relapsed SCLC but unfortunately did not demonstrate efficacy. A randomized phase II trial (NCT04768296) is assessing the efficacy of the ATR inhibitor Berzosertib in conjunction with topotecan for the treatment of relapsed, platinum-resistant SCLC, with the release of data anticipated. Concurrently Berzosertib conjugated with lurbinectedin (NCT04802174), as well as TROP-2 ADC (NCT0482634) clinical studies are still underway. Preclinical studies also indicate that CHK1 inhibitors, when combined with DNA-damaging chemotherapy, enhance the cytotoxic effects in SCLC models. Moreover, inhibition of CHK1 or PARP has been found to increase the levels of tumor-infiltrating T lymphocytes and synergize with anti-PD-L1 therapy, suggesting that DDR inhibitor treatment could enhance the efficacy of ICIs in SCLC patients^88^. Finally, the combination of PARP inhibitors with CHK1 inhibitors presents a convincing strategy to overcome PARP inhibitor resistance caused by replication fork stability and enhance therapeutic efficacy.

### 6.3 BCL-2 inhibitors

The BCL-2 family of proteins is mainly involved in mediating apoptosis, and it has also been observed that BCL-2 is widely expressed in SCLC and correlates with poor prognosis. Therapeutic strategies against BCL-2 targets are equally promising, yet agents targeting BCL-2 such as Navitoclax exhibited merely limited activity in single-agent trials against advanced and relapsed SCLC, with considerable side effects observed in some patients^89^, ^90^. Among the molecular subtypes of SCLC, BCL-2 inhibitors may be a therapeutic option for targeting the SCLC-A subtype.

### 6.4 Vaccines

Vaccines are potent in spurring the immune system to recognize and target specific tumor-derived neoantigens for action. Vaccines against tumors act by introducing tumor antigens or antigen pools as well as immunostimulatory vectors that sensitize the host's T-cells and drive a cytotoxic response against the aberrant cells. Integrating labeled tumor antigens or pools of candidate antigens to generate specific targeted neoantigens in tumors may be an efficacious modality for eliminating residual disease and improving antitumor efficacy. BEC2 vaccination in the SCLC treatment paradigm, while yielding a long median relapse-free survival for SCLC patients who achieved complete remission in individual studies, the subsequent phase III clinical trials failed to confer a significant benefit in terms of OS and PFS. Similarly, the development of vaccines such as 1E10 and Fuc-GM1 has been limited by factors related to efficacy assessment and side effects. An anti-tumor vaccine targeting the dendritic cell composition of P53 with a new paradigm of combination chemotherapy demonstrated high anti-tumor efficacy, providing a renewed reference. Overall, the efficacy of current tumor vaccine studies performed in SCLC patients awaits further verification, but vaccination of patients with individual tumor mutations may pose as a consequential therapeutic option.

### 6.5 Oncolytic virus

The principle of oncolytic virus therapy is to utilize replicating viruses to combat malignant tumors, and therefore, oncolytic virus therapy represents a potential therapeutic approach for tumor treatment^91^, ^92^. With the use of the Coxsackie adenovirus receptor at high tumor expression levels in clinical trials related to SCLC treatment recently, a recombinant Coxsackie virus B3 (CVB3) has been exploited to develop a recombinant Coxsackie virus B3, which has potent antiviral efficacy for TP53/RB1 mutant SCLC while minimizing the toxicity to normal tissues. Note on CVB3 genome as RNA viruses, their stability is inferior to that of DNA viruses. Neckar Valley virus (SVV) (NTX-010) is a novel natural oncolytic RNA virus in the small RNA virus genus with strong activity and selective tropism for SCLC. During a phase II clinical study conducted in patients treated with chemotherapy followed by non-progression disease, that no benefit in terms of PFS and OS was observed in the group treated with NTX-010 versus placebo.^93^ Later investigators developed a modified mucosal tumor-lytic tumor virus (MYXV) by displaying a tendency for moderate antitumor activity and safety in mice model. Besides, it was revealed that the infiltration of lysoviral activity by inducing immune cells showed signals that may have the potential to enhance the immunogenicity of tumors and increase the response rate to immunotherapy. Emerging therapies involving antiangiogenic classes, complex kinase inhibitors, and bispecific T-cell bridging antibodies have shown encouraging efficacy. Finally, other strategies including those targeting agents such as c-kit receptor, insulin-like growth factor receptor and Cellular-Mesenchymal-Epithelial Transition Factor inhibitors possibly could be a treatment option for SCLC, although further confirmation from real-world research data remains pending. Briefly, SCLC precision medicine strategies are being steadily developed, and the exploitation of novel targets has the advantage of better pharmacological properties and targeting for overcoming the effectiveness of SCLC drug resistance, thus expanding the limitations of SCLC treatment strategies and improving the competitiveness of clinical applications. However, the current anti-tumor efficacy for SCLC is still confronted with obstacles, leaving a certain distance from clinical application.

## 7. Future directions

An ongoing challenge for clinical management is the rampant development of therapeutic resistance and the scarcity of customized precision treatments following resistance to existing therapies. Determining the subcategorization respective therapeutic vulnerability exploring and elucidating the clinical relevance of SCLC transcriptional subtypes are future research hotspots as the initial framework for molecular phenotyping of SCLC emerges. Meanwhile, future work will also be embarked on leveraging multi-omics technologies, liquid biopsy techniques to investigate the molecular biological mechanisms of SCLC to better understand the impact of therapeutic fragility and resistance to therapeutics. Additionally, epigenetic regulators (e.g., LSD1 and EZH2) may have potentially potent functions in plastic changes in NE differentiation, and while preliminary clinical signals have yet to be observed, further efforts will need to be focused on the impact of epigenetic regulatory mechanisms on therapeutic resistance as an efficacy practice in the future. Ultimately, given the great promise of impressive profit from DLL3-targeted therapies for SCLC, additional exploration and validation of therapeutic approaches that integrate complementary pathways and diversity of clinical trials are promising for improving outcomes. To conclude, future management of SCLC shall be focused on exploiting precision medicine interventions through biomarker screening pertinent to the treatment fragility of specific patients. Simultaneous work on the development of novel therapeutics and integration of existing therapeutic regimes will be crucial strategies for combating drug resistance, whilst optimization of the whole management paradigm underpins the invaluable effort for improved survival in SCLC patients.

## 8. Conclusions

SCLC is an aggressive malevolent cancer harboring a dismal overall prognosis. Prior to the emergence of multiple targeted therapies against non-small cell lung cancer recently, there are currently unavailable potent targets or targeted regimens for SCLC. Complementation of immunotherapies in attempting towards exploration and optimization of the long-standing therapeutic status quo in SCLC, though the survivorship benefit persists to elude satisfactory achievement. Remarkable strides in identifying effective immune biomarkers and exploration of novel therapeutic modalities for SCLC have not yet been achieved up to this point. Nevertheless, the deepening comprehension of SCLC molecular phenotyping and genome-wide profiling over the years allowed the formulation of a preliminary framework for SCLC molecular phenotyping, identification of potentially related biomarkers for predicting the therapeutic efficacy of SCLC, and determination of potential targets including DLL3, thereby accomplishing long-awaited advances in understanding and therapies for SCLC, along with the simultaneous acquisition and optimization of novel therapies (e.g., CAR T-cell regimens) will hopefully further augment the sustained response and resistance of SCLC tumors and ultimately improved the survival outcomes of SCLC patients.

## Figures and Tables

**Table 1:** Overview of clinical study outcomes related to ICIs for extensive-stage SCLC.

Treatment	Clinical Trial	Grade≥ 3 TRAE	Outcomes	
			Median PFS (months)	Median OS (months)
First-line treatment for SCLC				
Impower133b (Phase III)	Atezolizumab + Chemo. *vs.* Chemo. + placebo	58.6%* vs.* 57.6%	5.2 *vs.* 4.3 (HR: 0.77, *P* = 0.02)	12.3* vs.* 10.3 (HR: 0.70, *P* = 0.007)
CASPIAN (Phase III)	1.Durvalumab + Tremelimumab + Chemo. *vs.* 2. Durvalumab + Chemo. *vs.* 3. Chemo.	73.0% *vs.* 65.0%* vs.* 65.0%	4.9 *vs.* 5.1 *vs.* 5.4; 1 *vs.* 3 (HR:0.84, *P* >0.05) 2 *vs.* 3 (HR:0.78, *P* >0.05)	10.4 *vs.*12.9 *vs.*10.5 1 *vs.* 3 (HR:0.82, *P* = 0.045) 2 *vs.* 3 (HR:0.75, *P* = 0.003)
CA184-156 (Phase III)	Ipilimumab + Chemo. *vs.* Chemo. + placebo	48.3%* vs.* 45.0%	4.6 *vs.* 4.4 (HR: 0.85, *P* = 0.016)	11* vs.* 10.9 (HR: 0.94, *P* = 0.378)
KEYNOTE-604 (Phase III)	Pembrolizumab+ Chemo. *vs.* Chemo. + placebo	83.0%* vs.* 80.3%	4.5 *vs.* 4.3 (HR: 0.75, *P* = 0.002)	10.8* vs.* 9.7 (HR: 0.8, *P* = 0.016)
ECOG-ACRIN-EA5161 (Phase II)	Nivolumab + Chemo. *vs*. Chemo.	77.0%* vs.* 62.0%	5.5 *vs.* 4.7 (HR: 0.68, *P* = 0.047)	11.3* vs.* 8.5 (HR: 0.67, *P* = 0.038)
(CAPSTONE-1) (Phase III)	Adebrelimab + Chemo. *vs.* Chemo. + placebo	85.7%* vs.* 84.9%	5.8 *vs.* 5.6 (HR: 0.67, *P* < 0.001)	15.3* vs.* 12.8 (HR: 0.72, *P* = 0.002)
(ASTRUM-005) (Phase III)	Serplulimab + Chemo. *vs.* Chemo. + placebo	33.2%* vs.* 27.6%	5.7 *vs.* 4.3 (HR: 0.48, *P* < 0.001)	15.4* vs.* 10.9 (HR: 0.63, *P* < 0.001)
SKYSCRAPER-02) (Phase III)	Atezolizumab + Tiragolumab + Chemo. *vs* atezolizumab + Chemo. + placebo	52.7%* vs.* 57.7%	5.4 *vs.* 5.6 (HR: 1.11, *P* = 0.350)	13.6* vs.* 13.6 (HR: 1.04, *P* = 0.796)
RATIONALE-312 (Phase III)	Tislelizumab + Chemo. *vs.* Chemo. + placebo	88.5% *vs.* 90.0%	4.8 *vs.* 4.3 (HR: 0.63, *P* < 0.001)	15.5 *vs.* 13.5 (HR: 0.75, *P* = 0.004)
EXTENTORCH (Phase III)	Toripalimab + Chemo. *vs.* Chemo. + placebo	89.6% *vs.* 89.4%	5.8 *vs.* 5.6 (HR: 0.667, *P* < 0.001)	14.6 *vs.* 13.3 (HR: 0.798, *P* = 0.033)
ETER701 (Phase III)	Benmelstobart + Anlotinib + Chemo. *vs.* 2. Anlotinib + Chemo. *vs.* 3. Chemo.	93.1% *vs.* 94.3%* vs.* 87.0%	6.9 *vs.* 5.6 *vs.* 4.2; 1 *vs.* 3 (HR:0.32, *P* < 0.001) 2 *vs.* 3 (HR:0.44,* P* < 0.001)	19.3 *vs.*12.9 *vs.*11.9 1 *vs.* 3 (HR:0.61, *P* = 0.0002) 2 *vs.* 3 (HR:0.86, *P* = 0.1723)
Backline treatment for SCLC				
CheckMate 33 (Phase III)	Nivolumab *vs*. Chemo.	13.8% *vs.* 73.2%	1.4 *vs.* 3.8 (HR: 1.41, *p* not test)	7.5 *vs.* 8.4 (HR: 0.86, *P* = 0.11)
IFCT-1603 (Phase II)	Atezolizumab *vs*. Chemo.	4.2% *vs.* 75.0%	1.4 *vs.* 4.3 (HR: 2.26, *P* = 0.004)	9.5 *vs.* 8.7 (HR: 0.86, *P* = 0.11)
KEYNOTE-158 (Phase II)	Pembrolizumab	-	2.0	9.1
BALTIC (Phase II)	Durvalumab + Tremelimumab	47.6%	1.9	6.0
KEYNOTE-028 (Phase Ib)	Pembrolizumab	33.3	1.9	9.7
CheckMate 032 (Phase I/II)	1. Nivolumab, 2. Nivolumab + Ipilimumab	12.9% *vs.* 37.5%	1:1.4, 2: 1.5	1:5.6, 2:5.7
ALTER 1202 (Phase II)	Anlotinib* vs*. placebo	35.8% *vs.* 15.4%	4.1* vs.* 0.7 (HR: 0.19,* P* < 0.001)	7.3* vs.* 4.9 (HR: 0.53,* P* = 0.0029)

Abbreviation: ICIs: Immune checkpoint inhibitors; SCLC: Small Cell Lung Cancer; Chemo.: Chemotherapy; PFS: Progression Free Survival; OS: Overall Survival;* vs.*: versus; HR: Hazard Ratio; TRAE: Treatment-Related Adverse Event.

**Table 2:** Clinical Trial Roundup for DLL3-Targeted SCLC Therapies.

Therapeutic drug	Mechanism of action	Clinical Trial Status	Indications	Key Results
**ADC-based drugs**				
Rovalpituzumab tesirine	Targeting DLL3 ADC	Phase I (NCT02674568^77^) (NCT01901653^74^) (NCT02874664*) (NCT03061812^94^) (NCT03086239^95^)	Relapsed/extensive/advanced SCLC/DLL3 expressing SCLC	Median OS (month): Overall group: 5.6; DLL3-high subgroup: 5.7 ORR: 11/60 (18%) DLL3-high: 10/26 (38%) - - -
SC-002	Targeting DLL3 ADC	Phase I (NCT02500914^76^)	Recurrent SCLC or large cell neuroendocrine carcinoma	ORR: 5/35 (14%) Grade ≥3 AEs (%): 24/35 (69%)
**CAR T drugs**				
AMG 119	Anti-DLL3 transduced autologous T cells	Phase I (suspended) (NCT03392064)	Relapsed/refractory SCLC	-
LB2102	Targeting DLL3 Dual Epitope CAR-T	Phase I (Recruiting) (NCT05680922) (NCT05620342)	Extensive stage SCLC or large cell neuroendocrine carcinoma	- - -
NK cell	DLL3 CAR NK cells	Phase I (Unknown) (NCT05507593)	Relapsed/refractory extensive-stage SCLC	-
**Specific Antibodies**				
Tarlatamab (AMG-757)	DLL3/CD3 T-cell Splicer Antibody	Phase II (NCT03319940^96^) (NCT05060016^24^)	Relapsed/refractory SCLC	ORR: 25%; OS (months):17.5; DOR (months): 11.2 ORR: 10-mg group:22/40 (55%) 100-mg group: 16/28 (57%)
BI 764532	DLL3/CD3 T cell junction bispecific antibody	Phase I (NCT04429087^97^)	Refractory expression of DLL3 SCLC and other neuroendocrine tumors	-
HPN328	Tri-specific recombinant protein constructs	Phase I/2 (Recruiting) (NCT04471727)	Relapsed/refractory advanced malignant tumors expressing DLL3	-
RO7616789	DLL3/ CD3/CD137 multi-specific antibody	Phase I (Recruiting) (NCT05619744)	Relapsed extensive stage SCLC or neuroendocrine carcinomas of either origin	-

SCLC: Small Cell Lung Cancer; DLL3: Delta-like ligand 3; NCT: National Clinical Trial; ADC: Antibody-drug conjugates receptor; CAR T: Chimeric antigen T; TriTAC: Tri-specific T cell activating construct; NK cell: Natural killer cell; AEs: Adverse events; OS: Overall survival; ORR: Objective remission rates; DOR: Duration of response; *: Not provided.

## Abbreviations

SCLC: Small cell lung cancer
DLL3: Delta-like ligand 3
CAR T: Chimeric antigen T
ADC: Antibody-drug conjugates receptor
TNM: tumor-nodes-metastasis
LS: limited-stage
ES: extensive-stage
ICIs: iImmune checkpoint inhibitors
PD-1: Programmed death-1
PD-L1: Programmed death ligand 1
ORR: Objective remission rates
OS: Overall Survival
TMB: Tumor mutational burden
mut/Mb: Mutations per megabase
NE: Neuroendocrine
TILs: Tumor-infiltrating lymphocytes
PNS: Paraneoplastic neurological syndrome
MHC: Major histocompatibility complex
ASCL1: Achaete-scute homolog 1
HLA: Hman leukocyte antigen
EMT: Epithelial mesenchymal transition
SLFN11: Schlafen family member 11
BCL-2: B-cell lymphoma 2
PARP: Poly ADP-ribose polymerase
NCT: National Clinical Trial
TME: Tumor microenvironment
OTOT: Off-tumor toxicity
ITAMs: Immunoreceptor tyrosine activation motif sequences
Rova-T: Rovalpituzumab tesirine
BiTEs: Bispecific T-cell Engagers
TriTAC: Tri-specific T cell activating construct
DDR: DNA Damage Response
PDX: Patient-derived xenograft
ATR: Ataxia Telangiectasia and Rad3
CHK1: Checkpoint kinase 1
CVB3: Coxsackie virus B3

## References

[1] Guan X, Liang J, Xiang Y, Li T, Zhong X (2024). BARX1 repressed FOXF1 expression and activated Wnt/β-catenin signaling pathway to drive lung adenocarcinoma. Int J Biol Macromol.

[2] Guo Q, Li D, Luo X (2021). The Regulatory Network and Potential Role of LINC00973-miRNA-mRNA ceRNA in the Progression of Non-Small-Cell Lung Cancer. Front Immunol.

[3] Tian Y, Xin S, Wan Z (2024). TCF19 promotes cell proliferation and tumor formation in lung cancer by activating the Raf/MEK/ERK signaling pathway. Transl Oncol.

[4] Yao Y, Guan X, Bao G, Liang J, Li T, Zhong X (2022). Whole-exome sequencing and bioinformatics analysis of a case of non-alpha-fetoprotein-elevated lung hepatoid adenocarcinoma. Front Pharmacol.

[5] Zeng L, Liang L, Fang X (2023). Glycolysis induces Th2 cell infiltration and significantly affects prognosis and immunotherapy response to lung adenocarcinoma. Funct Integr Genomics.

[6] Guo W, Qiao T, Li T (2022). The role of stem cells in small-cell lung cancer: evidence from chemoresistance to immunotherapy. Semin Cancer Biol.

[7] Li T, Qiao T (2022). Unraveling tumor microenvironment of small-cell lung cancer: Implications for immunotherapy. Semin Cancer Biol.

[8] Gazdar AF, Bunn PA, Minna JD (2017). Small-cell lung cancer: what we know, what we need to know and the path forward. Nat Rev Cancer.

[9] Govindan R, Page N, Morgensztern D (2006). Changing epidemiology of small-cell lung cancer in the United States over the last 30 years: analysis of the surveillance, epidemiologic, and end results database. J Clin Oncol.

[10] Rudin CM, Brambilla E, Faivre-Finn C, Sage J (2021). Small-cell lung cancer. Nat Rev Dis Primers.

[11] Torre LA, Siegel RL, Jemal A (2016). Lung Cancer Statistics. Adv Exp Med Biol.

[12] Hovanec J, Siemiatycki J, Conway DI (2018). Lung cancer and socioeconomic status in a pooled analysis of case-control studies. PLoS One.

[13] Stram DO, Park SL, Haiman CA (2019). Racial/Ethnic Differences in Lung Cancer Incidence in the Multiethnic Cohort Study: An Update. J Natl Cancer Inst.

[14] Zhou K, Shi H, Chen R (2021). Association of Race, Socioeconomic Factors, and Treatment Characteristics With Overall Survival in Patients With Limited-Stage Small Cell Lung Cancer. JAMA Netw Open.

[15] Wang S, Tang J, Sun T (2017). Survival changes in patients with small cell lung cancer and disparities between different sexes, socioeconomic statuses and ages. Sci Rep.

[16] Yang S, Zhang Z, Wang Q (2019). Emerging therapies for small cell lung cancer. J Hematol Oncol.

[17] Cheng Y, Spigel DR, Cho BC (2024). Durvalumab after Chemoradiotherapy in Limited-Stage Small-Cell Lung Cancer. N Engl J Med.

[18] Horn L, Mansfield AS, Szczęsna A (2018). First-Line Atezolizumab plus Chemotherapy in Extensive-Stage Small-Cell Lung Cancer. N Engl J Med.

[19] Paz-Ares L, Dvorkin M, Chen Y (2019). Durvalumab plus platinum-etoposide versus platinum-etoposide in first-line treatment of extensive-stage small-cell lung cancer (CASPIAN): a randomised, controlled, open-label, phase 3 trial. Lancet.

[20] Cheng Y, Chen J, Zhang W (2024). Benmelstobart, anlotinib and chemotherapy in extensive-stage small-cell lung cancer: a randomized phase 3 trial. Nat Med.

[21] Lee WS, Yang H, Chon HJ, Kim C (2020). Combination of anti-angiogenic therapy and immune checkpoint blockade normalizes vascular-immune crosstalk to potentiate cancer immunity. Exp Mol Med.

[22] Syed YY (2018). Anlotinib: First Global Approval. Drugs.

[23] Eckardt JR, von Pawel J, Pujol JL (2007). Phase III study of oral compared with intravenous topotecan as second-line therapy in small-cell lung cancer. J Clin Oncol.

[24] Ahn MJ, Cho BC, Felip E (2023). Tarlatamab for Patients with Previously Treated Small-Cell Lung Cancer. N Engl J Med.

[25] Chung HC, Piha-Paul SA, Lopez-Martin J (2020). Pembrolizumab After Two or More Lines of Previous Therapy in Patients With Recurrent or Metastatic SCLC: Results From the KEYNOTE-028 and KEYNOTE-158 Studies. J Thorac Oncol.

[26] Ready N, Farago AF, de Braud F (2019). Third-Line Nivolumab Monotherapy in Recurrent SCLC: CheckMate 032. J Thorac Oncol.

[27] Cheng Y, Wang Q, Li K (2021). Anlotinib vs placebo as third- or further-line treatment for patients with small cell lung cancer: a randomised, double-blind, placebo-controlled Phase 2 study. Br J Cancer.

[28] Ye L, Creaney J, Redwood A, Robinson B (2021). The Current Lung Cancer Neoantigen Landscape and Implications for Therapy. J Thorac Oncol.

[29] Cheng Y, Fan Y, Zhao Y (2024). Tislelizumab Plus Platinum and Etoposide Versus Placebo Plus Platinum and Etoposide as First-Line Treatment for Extensive-Stage SCLC (RATIONALE-312): A Multicenter, Double-Blind, Placebo-Controlled, Randomized, Phase 3 Clinical Trial. J Thorac Oncol.

[30] Liu SV, Reck M, Mansfield AS (2021). Updated Overall Survival and PD-L1 Subgroup Analysis of Patients With Extensive-Stage Small-Cell Lung Cancer Treated With Atezolizumab, Carboplatin, and Etoposide (IMpower133). J Clin Oncol.

[31] Rudin CM, Awad MM, Navarro A (2020). Pembrolizumab or Placebo Plus Etoposide and Platinum as First-Line Therapy for Extensive-Stage Small-Cell Lung Cancer: Randomized, Double-Blind, Phase III KEYNOTE-604 Study. J Clin Oncol.

[32] Pleasance ED, Stephens PJ, O'Meara S (2010). A small-cell lung cancer genome with complex signatures of tobacco exposure. Nature.

[33] Peifer M, Fernández-Cuesta L, Sos ML (2012). Integrative genome analyses identify key somatic driver mutations of small-cell lung cancer. Nat Genet.

[34] Hanglow AC, Perdue MH, Dyck N, Bienenstock J (1988). Role of nonspecific cytotoxic cells in intestinal epithelial cell injury in Nippostrongylus brasiliensis infection. Clin Immunol Immunopathol.

[35] Yarchoan M, Hopkins A, Jaffee EM (2017). Tumor Mutational Burden and Response Rate to PD-1 Inhibition. N Engl J Med.

[36] Hellmann MD, Callahan MK, Awad MM (2018). Tumor Mutational Burden and Efficacy of Nivolumab Monotherapy and in Combination with Ipilimumab in Small-Cell Lung Cancer. Cancer Cell.

[37] Antonia SJ, López-Martin JA, Bendell J (2016). Nivolumab alone and nivolumab plus ipilimumab in recurrent small-cell lung cancer (CheckMate 032): a multicentre, open-label, phase 1/2 trial. Lancet Oncol.

[38] Wang J, Zhou C, Yao W (2022). Adebrelimab or placebo plus carboplatin and etoposide as first-line treatment for extensive-stage small-cell lung cancer (CAPSTONE-1): a multicentre, randomised, double-blind, placebo-controlled, phase 3 trial. Lancet Oncol.

[39] Gandara DR, Paul SM, Kowanetz M (2018). Blood-based tumor mutational burden as a predictor of clinical benefit in non-small-cell lung cancer patients treated with atezolizumab. Nat Med.

[40] Nguyen EM, Taniguchi H, Chan JM (2022). Targeting Lysine-Specific Demethylase 1 Rescues Major Histocompatibility Complex Class I Antigen Presentation and Overcomes Programmed Death-Ligand 1 Blockade Resistance in SCLC. J Thorac Oncol.

[41] Mahadevan NR, Knelson EH, Wolff JO (2021). Intrinsic Immunogenicity of Small Cell Lung Carcinoma Revealed by Its Cellular Plasticity. Cancer Discov.

[42] Zhu L, Qin J (2024). Predictive biomarkers for immunotherapy response in extensive-stage SCLC. J Cancer Res Clin Oncol.

[43] Iams WT, Shiuan E, Meador CB (2019). Improved Prognosis and Increased Tumor-Infiltrating Lymphocytes in Patients Who Have SCLC With Neurologic Paraneoplastic Syndromes. J Thorac Oncol.

[44] Herbreteau G, Langlais A, Greillier L (2020). Circulating Tumor DNA as a Prognostic Determinant in Small Cell Lung Cancer Patients Receiving Atezolizumab. J Clin Med.

[45] George J, Lim JS, Jang SJ (2015). Comprehensive genomic profiles of small cell lung cancer. Nature.

[46] Gazdar AF, Carney DN, Nau MM, Minna JD (1985). Characterization of variant subclasses of cell lines derived from small cell lung cancer having distinctive biochemical, morphological, and growth properties. Cancer Res.

[47] Sutherland KD, Ireland AS, Oliver TG (2022). Killing SCLC: insights into how to target a shapeshifting tumor. Genes Dev.

[48] Reguart N, Marin E, Remon J, Reyes R, Teixido C (2020). In Search of the Long-Desired 'Copernican Therapeutic Revolution' in Small-Cell Lung Cancer. Drugs.

[49] Rudin CM, Poirier JT, Byers LA (2019). Molecular subtypes of small cell lung cancer: a synthesis of human and mouse model data. Nat Rev Cancer.

[50] Baine MK, Hsieh MS, Lai WV (2020). SCLC Subtypes Defined by ASCL1, NEUROD1, POU2F3, and YAP1: A Comprehensive Immunohistochemical and Histopathologic Characterization. J Thorac Oncol.

[51] Gay CM, Stewart CA, Park EM (2021). Patterns of transcription factor programs and immune pathway activation define four major subtypes of SCLC with distinct therapeutic vulnerabilities. Cancer Cell.

[52] Byers LA, Diao L, Wang J (2013). An epithelial-mesenchymal transition gene signature predicts resistance to EGFR and PI3K inhibitors and identifies Axl as a therapeutic target for overcoming EGFR inhibitor resistance. Clin Cancer Res.

[53] Horvath L, Lang C, Boettiger K, Aigner C, Dome B, Megyesfalvi Z (2024). Potential subtype-specific therapeutic approaches in small cell lung cancer. Curr Opin Oncol.

[54] Schwendenwein A, Megyesfalvi Z, Barany N (2021). Molecular profiles of small cell lung cancer subtypes: therapeutic implications. Mol Ther Oncolytics.

[55] Owonikoko TK, Dahlberg SE, Sica GL (2019). Randomized Phase II Trial of Cisplatin and Etoposide in Combination With Veliparib or Placebo for Extensive-Stage Small-Cell Lung Cancer: ECOG-ACRIN 2511 Study. J Clin Oncol.

[56] Angelopoulou A, Theocharous G, Valakos D (2024). Loss of the tumour suppressor LKB1/STK11 uncovers a leptin-mediated sensitivity mechanism to mitochondrial uncouplers for targeted cancer therapy. Mol Cancer.

[57] Chen J, Guanizo AC, Jakasekara W (2023). MYC drives platinum resistant SCLC that is overcome by the dual PI3K-HDAC inhibitor fimepinostat. J Exp Clin Cancer Res.

[58] Tong Q, Ouyang S, Chen R, Huang J, Guo L (2019). MYCN-mediated regulation of the HES1 promoter enhances the chemoresistance of small-cell lung cancer by modulating apoptosis. Am J Cancer Res.

[59] Lim JS, Ibaseta A, Fischer MM (2017). Intratumoural heterogeneity generated by Notch signalling promotes small-cell lung cancer. Nature.

[60] Wu Q, Guo J, Liu Y (2021). YAP drives fate conversion and chemoresistance of small cell lung cancer. Sci Adv.

[61] Ireland AS, Micinski AM, Kastner DW (2020). MYC Drives Temporal Evolution of Small Cell Lung Cancer Subtypes by Reprogramming Neuroendocrine Fate. Cancer Cell.

[62] Sabari JK, Lok BH, Laird JH, Poirier JT, Rudin CM (2017). Unravelling the biology of SCLC: implications for therapy. Nat Rev Clin Oncol.

[63] Saunders LR, Bankovich AJ, Anderson WC (2015). A DLL3-targeted antibody-drug conjugate eradicates high-grade pulmonary neuroendocrine tumor-initiating cells in vivo. Sci Transl Med.

[64] Borromeo MD, Savage TK, Kollipara RK (2016). ASCL1 and NEUROD1 Reveal Heterogeneity in Pulmonary Neuroendocrine Tumors and Regulate Distinct Genetic Programs. Cell Rep.

[65] Schuster SJ, Svoboda J, Chong EA (2017). Chimeric Antigen Receptor T Cells in Refractory B-Cell Lymphomas. N Engl J Med.

[66] Lee JH, Saxena A, Giaccone G (2023). Advancements in small cell lung cancer. Semin Cancer Biol.

[67] Owen DH, Giffin MJ, Bailis JM, Smit MD, Carbone DP, He K (2019). DLL3: an emerging target in small cell lung cancer. J Hematol Oncol.

[68] Liu M, Huang W, Guo Y (2022). CAR NK-92 cells targeting DLL3 kill effectively small cell lung cancer cells in vitro and in vivo. J Leukoc Biol.

[69] Yarmarkovich M, Marshall QF, Warrington JM (2021). Cross-HLA targeting of intracellular oncoproteins with peptide-centric CARs. Nature.

[70] Williams JZ, Allen GM, Shah D (2020). Precise T cell recognition programs designed by transcriptionally linking multiple receptors. Science.

[71] Flugel CL, Majzner RG, Krenciute G (2023). Overcoming on-target, off-tumour toxicity of CAR T cell therapy for solid tumours. Nat Rev Clin Oncol.

[72] Drago JZ, Modi S, Chandarlapaty S (2021). Unlocking the potential of antibody-drug conjugates for cancer therapy. Nat Rev Clin Oncol.

[73] Belluomini L, Sposito M, Avancini A (2023). Unlocking New Horizons in Small-Cell Lung Cancer Treatment: The Onset of Antibody-Drug Conjugates. Cancers (Basel).

[74] Rudin CM, Pietanza MC, Bauer TM (2017). Rovalpituzumab tesirine, a DLL3-targeted antibody-drug conjugate, in recurrent small-cell lung cancer: a first-in-human, first-in-class, open-label, phase 1 study. Lancet Oncol.

[75] Johnson ML, Zvirbule Z, Laktionov K (2021). Rovalpituzumab Tesirine as a Maintenance Therapy After First-Line Platinum-Based Chemotherapy in Patients With Extensive-Stage-SCLC: Results From the Phase 3 MERU Study. J Thorac Oncol.

[76] Morgensztern D, Johnson M, Rudin CM (2020). SC-002 in patients with relapsed or refractory small cell lung cancer and large cell neuroendocrine carcinoma: Phase 1 study. Lung Cancer.

[77] Morgensztern D, Besse B, Greillier L (2019). Efficacy and Safety of Rovalpituzumab Tesirine in Third-Line and Beyond Patients with DLL3-Expressing, Relapsed/Refractory Small-Cell Lung Cancer: Results From the Phase II TRINITY Study. Clin Cancer Res.

[78] Blackhall F, Jao K, Greillier L (2021). Efficacy and Safety of Rovalpituzumab Tesirine Compared With Topotecan as Second-Line Therapy in DLL3-High SCLC: Results From the Phase 3 TAHOE Study. J Thorac Oncol.

[79] Malhotra J, Nikolinakos P, Leal T (2021). A Phase 1-2 Study of Rovalpituzumab Tesirine in Combination With Nivolumab Plus or Minus Ipilimumab in Patients With Previously Treated Extensive-Stage SCLC. J Thorac Oncol.

[80] Giffin MJ, Cooke K, Lobenhofer EK (2021). AMG 757, a Half-Life Extended, DLL3-Targeted Bispecific T-Cell Engager, Shows High Potency and Sensitivity in Preclinical Models of Small-Cell Lung Cancer. Clin Cancer Res.

[81] Paz-Ares L, Champiat S, Lai WV (2023). Tarlatamab, a First-in-Class DLL3-Targeted Bispecific T-Cell Engager, in Recurrent Small-Cell Lung Cancer: An Open-Label, Phase I Study. J Clin Oncol.

[82] AMGEN (2024). AMGEN PRESENTS AMGEN PRESENTS NEW DATA FOR FIRST-IN-CLASS IMDELLTRA™ (TARLATAMAB-DLLE) IN SMALL CELL LUNG CANCER AT WCLC 2024. https://www.amgen.com/newsroom/press-releases/2024/09/amgen-presents-new-data-for-firstinclass-imdelltra-tarlatamabdlle-in-small-cell-lung-cancer-at-wclc-2024. Retrieved September 10.

[83] Hipp S, Voynov V, Drobits-Handl B (2020). A Bispecific DLL3/CD3 IgG-Like T-Cell Engaging Antibody Induces Antitumor Responses in Small Cell Lung Cancer. Clin Cancer Res.

[84] Martin Wermke EF, Yasutoshi Kuboki DM, Cyrus Sayehli MFS, Edurne Arriola ZO, Eric Song MS, Gambardella aV (2023). First-in-human dose-escalation trial of BI 764532, a delta-like ligand 3 (DLL3)/CD3 IgG like T-cell engager in patients (pts) with DLL3-positive (DLL3+) small-cell lung cancer(SCLC) and neuroendocrine carcinoma (NEC). JOURNAL OF CLINICAL ONCOLOGY.

[85] Byers LA, Wang J, Nilsson MB (2012). Proteomic profiling identifies dysregulated pathways in small cell lung cancer and novel therapeutic targets including PARP1. Cancer Discov.

[86] Pietanza MC, Waqar SN, Krug LM (2018). Randomized, Double-Blind, Phase II Study of Temozolomide in Combination With Either Veliparib or Placebo in Patients With Relapsed-Sensitive or Refractory Small-Cell Lung Cancer. J Clin Oncol.

[87] Farago AF, Yeap BY, Stanzione M (2019). Combination Olaparib and Temozolomide in Relapsed Small-Cell Lung Cancer. Cancer Discov.

[88] Sen T, Rodriguez BL, Chen L (2019). Targeting DNA Damage Response Promotes Antitumor Immunity through STING-Mediated T-cell Activation in Small Cell Lung Cancer. Cancer Discov.

[89] Gandhi L, Camidge DR, Ribeiro de Oliveira M (2011). Phase I study of Navitoclax (ABT-263), a novel Bcl-2 family inhibitor, in patients with small-cell lung cancer and other solid tumors. J Clin Oncol.

[90] Rudin CM, Hann CL, Garon EB (2012). Phase II study of single-agent navitoclax (ABT-263) and biomarker correlates in patients with relapsed small cell lung cancer. Clin Cancer Res.

[91] Hemminki O, Dos Santos JM, Hemminki A (2020). Oncolytic viruses for cancer immunotherapy. J Hematol Oncol.

[92] Zhang Y, Li Y, Chen K, Qian L, Wang P (2021). Oncolytic virotherapy reverses the immunosuppressive tumor microenvironment and its potential in combination with immunotherapy. Cancer Cell Int.

[93] Schenk EL, Mandrekar SJ, Dy GK (2020). A Randomized Double-Blind Phase II Study of the Seneca Valley Virus (NTX-010) versus Placebo for Patients with Extensive-Stage SCLC (ES SCLC) Who Were Stable or Responding after at Least Four Cycles of Platinum-Based Chemotherapy: North Central Cancer Treatment Group (Alliance) N0923 Study. J Thorac Oncol.

[94] Tanaka K, Isse K, Fujihira T (2018). Prevalence of Delta-like protein 3 expression in patients with small cell lung cancer. Lung Cancer.

[95] Udagawa H, Akamatsu H, Tanaka K (2019). Phase I safety and pharmacokinetics study of rovalpituzumab tesirine in Japanese patients with advanced, recurrent small cell lung cancer. Lung Cancer.

[96] Dowlati A, Hummel HD, Champiat S (2024). Sustained Clinical Benefit and Intracranial Activity of Tarlatamab in Previously Treated Small Cell Lung Cancer: DeLLphi-300 Trial Update. J Clin Oncol.

[97] Wermke M, Felip E, Gambardella V (2022). Phase I trial of the DLL3/CD3 bispecific T-cell engager BI 764532 in DLL3-positive small-cell lung cancer and neuroendocrine carcinomas. Future Oncol.

